# Efficient Gene Expression System in Medaka Embryos Enables Functional Characterization of *nt5c1a* Paralogs Involved in Inosine Monophosphate Metabolism

**DOI:** 10.1002/dvg.70056

**Published:** 2026-05-09

**Authors:** Yu Murakami, Tomohisa Horibe, Masashi Ando, Toru Kobayashi

**Affiliations:** ^1^ Department of Fisheries Graduate School of Agriculture, Kindai University Nara Japan; ^2^ Department of Biological Data Science, Faculty of Bio‐Science Nagahama Institute of Bio‐Science and Technology Nagahama‐Shi Shiga Japan

**Keywords:** cytosolic 5′‐nucleotidase 1a (nt5c1a), inosine monophosphate (IMP), luciferase assay, medaka, microinjection, transient expression system

## Abstract

Optimizing transient expression systems in fish embryos is crucial for rapid gene function analysis. Here, we established an efficient system in medaka ( *Oryzias latipes* ) embryos by evaluating nucleic acid type and injection site. Our results revealed that injecting the *elongation factor 1αA* (*ef1αA*) promoter‐driven plasmid into the yolk yielded the highest expression on day 1 post‐fertilization. Using this optimized system, we investigated *cytosolic 5′‐nucleotidase 1a* (*nt5c1a*), which is involved in the metabolism of inosine monophosphate (IMP), an umami flavor compound. In silico analysis revealed that medaka had two *nt5c1a* paralogs: *nt5c1aa* and *nt5c1ab*. While *nt5c1ab* retains conserved substrate‐recognition sequences and exhibits significant IMP degradation activity, *nt5c1aa* has lost these functions. Structural analysis using AlphaFold revealed that the Nt5c1aa L305P mutation causes local conformational changes near the substrate‐binding site, potentially altering substrate orientation without disrupting the overall protein fold. Our expression system demonstrated that this single L305P substitution partially restored IMP‐degrading activity in Nt5c1aa, confirming that residue 305 is a key determinant of its functional divergence. Our findings provide a robust foundation for molecular breeding to enhance umami flavor in farmed fish. Specifically, the targeted manipulation of these *nt5c1a* paralogs could facilitate developing breeds with maximized IMP accumulation in muscle tissues.

## 1 Introduction

Aquaculture is a rapidly growing industry that has become the primary source of fish for human consumption (Verdegem et al. 2023). Many livestock species and agricultural crops have been subjected to selective breeding for human consumption for thousands of years; however, there remains much room for the genetic improvement of fish (Mittler and Blumwald 2010; Gjedrem 2012; Biscarini et al. 2015). For example, the Food and Agriculture Organization (FAO) has reported that more than 600 fish species are farmed worldwide, but less than 10% have been genetically improved using breeding programs (FAO 2022). To overcome this deficiency, much effort has been devoted to developing biotechnologies to promote the molecular breeding of farmed fish in recent decades. In particular, the CRISPR‐Cas9 system is an innovative genome editing tool that can be used to produce new breeds with useful traits, such as enhanced growth and disease resistance in cultured fish (Wang et al. 2025; Wang and Doudna 2023). However, genetic analysis of fishes has lagged far behind that of mammals such as humans and mice, which hampers CRISPR‐based breeding. Thus, the molecular mechanisms underlying the physiology, metabolism, and other survival functions and genes associated with economic traits in fishes remain to be elucidated.

Among potential molecular targets for improving these traits, enzymes involved in purine metabolism have received increasing attention. Molecules belonging to the 5′‐nucleotidase (Nt5) family are promising targets for the CRISPR‐based breeding (Bianchi and Spychala 2003). In particular, ecto‐5′‐nucleotidase (Nt5e) regulates purine metabolism by catalyzing the dephosphorylation of inosine monophosphate (IMP), a key intermediate in purine degradation (Hunsucker et al. 2005). IMP is not only a metabolic intermediate but also a major umami‐imparting compound that positively contributes to the taste of fish (Yamaguchi and Ninomiya 2000; Kurihara 2015; Hossain et al. 2024). Thus, the suppression of Nt5e activity is an attractive approach for maintaining IMP levels, which may aid the production of fish with superior umami. Indeed, our research group previously demonstrated that the disruption of *nt5e* function is effective for retaining IMP in postmortem muscles of medaka ( *Oryzias latipes* ) using the CRISPR‐Cas9 system (Murakami et al. 2022). Additionally, two non‐synonymous single‐nucleotide polymorphisms (SNPs) of Nt5e affect the amount of IMP and its degradation products in beef by regulating the enzymatic activity of Nt5e (Uemoto et al. 2017). Thus, the elucidation of the IMP degradation system is the key to producing IMP‐rich fish. However, there are far fewer studies on the molecules responsible for IMP degradation in fish compared with those on other organisms, such as mammals. For instance, cytosolic 5′‐nucleotidase 1a (Nt5c1a) degrades IMP in the cytoplasm of pigeons, rabbits, and humans (Hunsucker et al. 2001); however, there is a lack of basic insights into Nt5c1a in fish. Although previous studies based on biochemical approaches have confirmed IMP‐degrading activity in extracts from tissues such as skeletal muscle, liver, and kidney in several fish species (Itoh and Kimura 2002; Seki and Hamada‐Sato 2015), there remains no evidence that Nt5c1a itself degrades IMPs in fish. Therefore, we evaluated whether *nt5c1a* is involved in IMP degradation using medaka in this study. Medaka has favorable features as an experimental fish, such as high fecundity, rapid maturity, and the availability of inbred strains (Takeda and Shimada 2010; Wittbrodt et al. 2002). In addition, its whole genome and transcriptome have been sequenced, and reverse genetic approaches, such as transgenesis and genome editing, have been established (Ishikawa et al. 2022). Because of these advantages, we chose medaka for use in this study.

Previously, we developed a transient assay system using medaka embryos, which is suitable for analyzing IMP‐degrading activity at the molecular level (Murakami et al. 2022). We transiently overexpressed medaka Nt5e by injecting mRNA that was synthesized in vitro into the embryos. The extracts of embryos at 1 day post‐fertilization (dpf) markedly reduced exogenous IMP levels in a reaction solution, showing that Nt5e enhanced IMP degradation. This transient assay accurately assessed the amount of IMP degradation without the influence of endogenous IMP because embryos at 1 dpf did not contain IMP. However, the expression efficiency of the transient assay is not optimized. Although mRNA injection is generally preferred for its rapid and uniform expression, the introduction of plasmid DNA can also be considered as an alternative for specific applications. In medaka, several practical tools, such as *β‐actin* (*actb*) and *elongation factor 1αA* (*ef1αA*) promoter, can be used for DNA‐based overexpression in the embryos (Hamada et al. 1998; Kinoshita et al. 1999). Furthermore, the maize transposon system, *Ac*/*Ds*, was used to enhance the expression of exogenous DNA in medaka (Boon Ng and Gong 2011). However, there have been no studies comparing the expression levels of target proteins derived from introduced DNA and mRNA in fish embryos. Microinjection is a tedious and delicate procedure; therefore, it is necessary to establish a simple protocol to efficiently express target proteins with as little effort as possible. Thus, we examined the expression of proteins derived from transduced DNA or mRNA and temporal changes during development.

The site of microinjection is another important factor to consider when developing a transient assay system. In general, efficient transgenesis requires delivery of the injected solution into the cytoplasm. The optimal injection site for fish embryos depends on the species, due to differences in the nature of the yolk (Goto et al. 2019). For instance, both the cytoplasm and the yolk are efficient injection sites in zebrafish ( *Danio rerio* ) because the yolk is granular; therefore, the solution injected into the yolk will flow into the cytoplasm (Rosen et al. 2009; Quick et al. 2021). In contrast, the injection sites for medaka are usually limited to the cytoplasm (Murakami and Kinoshita 2018). The yolk of medaka has a single pouch‐like structure, and it is thought that the solutions injected into the yolk are unlikely to transfer to the cytoplasm (Iwamatsu 2004). However, we knew empirically that when DNA or mRNA encoding the *enhanced green fluorescent protein* (E*GFP*) gene is injected into the medaka yolk, green fluorescence can be observed in both the yolk and cytoplasm (Murakami, 2022). This prompted us to test the hypothesis that the medaka yolk is a suitable injection site for the transient expression of foreign genes. Injection into the yolk is simpler than injection into the cytoplasm because it does not require as delicate and laborious a procedure, such as the orientation of the cytoplasm toward an injection needle on an embryo holder (Murakami and Kinoshita 2018). However, it is unclear how much expression efficiency differs between the cytoplasm and yolk, and whether yolk injection can be used for transient expression in medaka.

The present study was carried out to increase the efficiency of the embryo‐based transient assay and to analyze the function of *nt5c1a* in medaka. We examined parameters controlling gene expression using a firefly luciferase (LUC) activity assay. Moreover, we cloned two *nt5c1a* genes in medaka and validated their IMP degradation activities using the optimized assay system.

## 2 Results

### 2.1 Establishment of a High‐Expression System in Medaka Embryos Through Optimization of Injection Parameters

**FIGURE 1:**
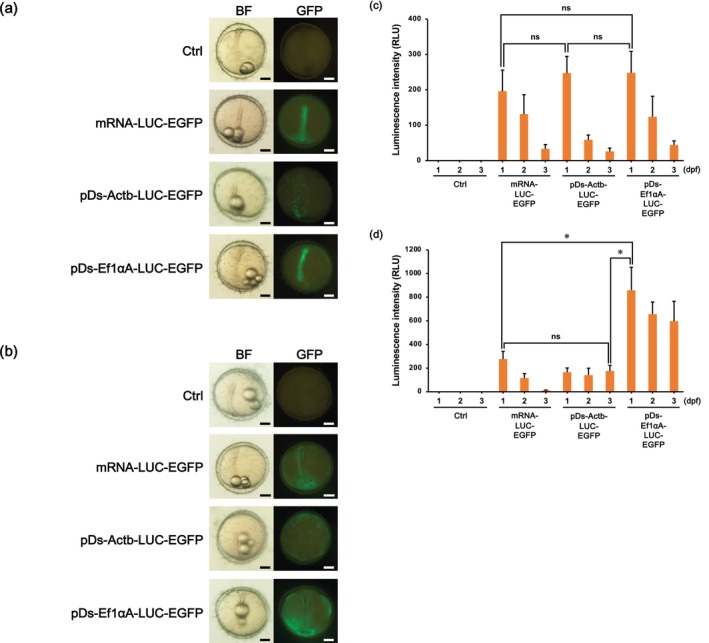
Expression of LUC‐EGFP reporter constructs following their microinjection into medaka embryos. (a) Representative fluorescence images of embryos at 1 day post‐ fertilization (dpf). Green fluorescence was detected in embryos that received injections into the cytoplasm of mRNA‐LUC‐GFP, pDs‐Actb‐LUC‐EGFP, or pDs‐Ef1αA‐LUC‐EGFP constructs. Embryos that did not receive microinjection were used as a control (Ctrl). Except for in the Ctrl, green fluorescence was mainly observed in the cytoplasm. (b) Representative fluorescence images of embryos that received injections into the yolk with the abovementioned four constructs. Except for in the Ctrl, green fluorescence signals were predominantly localized in the yolk. (a, b) BF, bright field; GFP, green fluorescent protein. Scale bars = 0.2 μm. (c) Quantitative comparison of luminescence intensity after cytoplasmic injection. Luminescence reached its highest level at 1 dpf in all the injected groups, with no significant difference among constructs. Signal intensity gradually decreased from 1 to 3 dpf. The control group (uninjected) showed no detectable luminescence. (d) Quantitative comparison of luminescence intensity after yolk injection. Injection of the pDs‐Ef1αA‐LUC‐EGFP construct resulted in the highest luminescence at 1 dpf, which was significantly greater than the maximum values observed in the other groups. The control group showed no detectable luminescence. (c, d) Data are presented as the mean ± SD of three independent experiments (*N* = 3), each performed using 12 embryos per group (*n* = 12). Statistical analyses were performed using one‐way ANOVA followed by Tukey's HSD test. Asterisks indicate significant differences (*p* < 0.05), and “ns” indicates no significant difference.

To establish an efficient method for transient expression assays, we constructed two plasmids containing the EGFP and LUC reporter genes and also synthesized mRNA encoding both these reporter genes (Figure S1a,b). Nucleic acid solutions containing either plasmid DNA or in vitro‐transcribed mRNA were injected into the cytoplasm of one‐cell stage embryos. Following injection, green fluorescence was clearly detected under a fluorescence microscope, confirming successful expression of the reporter constructs (Figure 1a). To quantitatively compare expression levels between the DNA‐ and mRNA‐based approaches, the luminescence intensity of the LUC reporter in each group was measured. As shown in Figure 1c, the expression levels were highest at 1 dpf in all injection groups injected into the cytoplasm and then decreased in a time‐dependent manner, indicating that transient expression peaks early in development. The maximum values for each group (mRNA‐LUC‐EGFP: 195.7 ± 59.5 relative luminescence units [RLU], pDs‐Actb‐LUC‐EGFP: 246.5 ± 47.6 RLU, and pDs‐Ef1αA‐LUC‐EGFP: 247.7 ± 55.2 RLU) were not significantly different, suggesting that both mRNA and DNA constructs provide comparable expression levels when targeting the cytoplasm. In contrast, changing the injection site from the cytoplasm to the yolk resulted in two major differences in the results. First, compared to injections into the cytoplasm, injections into the yolk resulted in weaker green fluorescence in the cytoplasm, while fluorescence in the yolk was stronger (Figure 1b). Second, the LUC reporter assay revealed that the group that received the Ef1αA promoter (pDs‐Ef1αA‐LUC‐EGFP: 859.5 ± 193.6 RLU) exhibited the highest fluorescence, which was significantly higher than that in the other groups (mRNA‐LUC‐EGFP: 277.3 ± 64.8 RLU and pDs‐Actb‐LUC‐EGFP: 165.1 ± 36.2 RLU) (Figure 1d). This demonstrates that the Ef1αA promoter is particularly effective for driving high‐level expression when delivered via yolk injection. In contrast to LUC quantification, EGFP‐based evaluation did not reveal significant differences among groups (Table 1). This discrepancy is due to the qualitative nature of the assay; embryos were classified as EGFP‐positive when any detectable fluorescence was observed, including weak signals. Notably, survival rates were significantly lower in the yolk injection group compared to the cytoplasmic injection group (Table 2). Based on these results, despite the reduction in viability, we adopted the technique of injecting a plasmid containing the Ef1αA promoter into the yolk for subsequent expression analysis to prioritize achieving the highest possible reporter activity.

**TABLE 1:** Results of microinjection and subsequent screening of founders.

Injected material	Injected site	No. of injected embryos	No. of survived embryos at 1dpf	Survival rate (%)	No. of expression embryos at 1 dpf	Expression rate (%)
Ctrl	ー	50	48	96.0	0	0.0
mRNA‐LUC‐EGFP	Cytoplasm	75	64	85.3	63	98.4
pDs‐Actb‐LUC‐EGFP	Cytoplasm	59	50	84.7	43	86.0
pDs‐Ef1αA‐LUC‐EGFP	Cytoplasm	64	51	79.7	49	96.1
mRNA‐LUC‐EGFP	Yolk	58	41	70.7	41	100.0
pDs‐Actb‐LUC‐EGFP	Yolk	73	47	64.4	45	95.7
pDs‐Ef1αA‐LUC‐EGFP	Yolk	62	43	69.4	42	97.7

*Note:* Survival rate (%) = No. of survived embryos at 1dpf/No. of injected embryos. Expression rate (%) = No. of expression embryos at 1 dpf/No. of survived embryos at 1dpf.

**TABLE 2:** Comparison of embryo survival rates between cytoplasmic and yolk injection.

Injected site	No. of injected embryos	No. of survived embryos at 1dpf	Survival rate (%)
Cytoplasm	198	165	83.3*
Yolk	193	131	67.9*

*Note:* Survival rate (%) = No. of survived embryos at 1dpf/No. of injected embryos. Data represent pooled counts from the three injected materials shown in Table 1 (mRNA‐LUC‐EGFP, pDs‐Actb‐LUC‐EGFP, and pDs‐Ef1αA‐LUC‐EGFP). The asterisk indicate that the survival rates of the yolk‐injected group are significantly lower than that of the cytoplasm‐injcted group according to Fisher's exact test (*p* < 0.05).

To further evaluate the suitability of plasmid injection conditions, we examined the dose dependency of plasmid concentration on embryo survival and transgene expression following yolk injection. Embryos were injected with a pDs‐Ef1αA‐LUC‐EGFP plasmid at concentrations of 1, 10, or 20 ng/μL, and survival rates were assessed at 1 dpf (Figure S2a). The dose–response analysis showed that while no significant difference in survival was observed between the 1 and 10 ng/μL groups, injection at concentrations of 20 ng/μL resulted in a significantly reduced survival rate. In parallel, LUC activity increased significantly at 10 ng/μL compared with 1 ng/μL, whereas no further increase was observed at 20 ng/μL (Figure S2b). These results indicate that plasmid injection at 10 ng/μL provides a suitable balance between high expression efficiency and acceptable levels of embryo survival; therefore, this concentration was used in subsequent experiments.

### 2.2 Identification and Characterization of Medaka nt5c1a Paralogs

**FIGURE 2:**
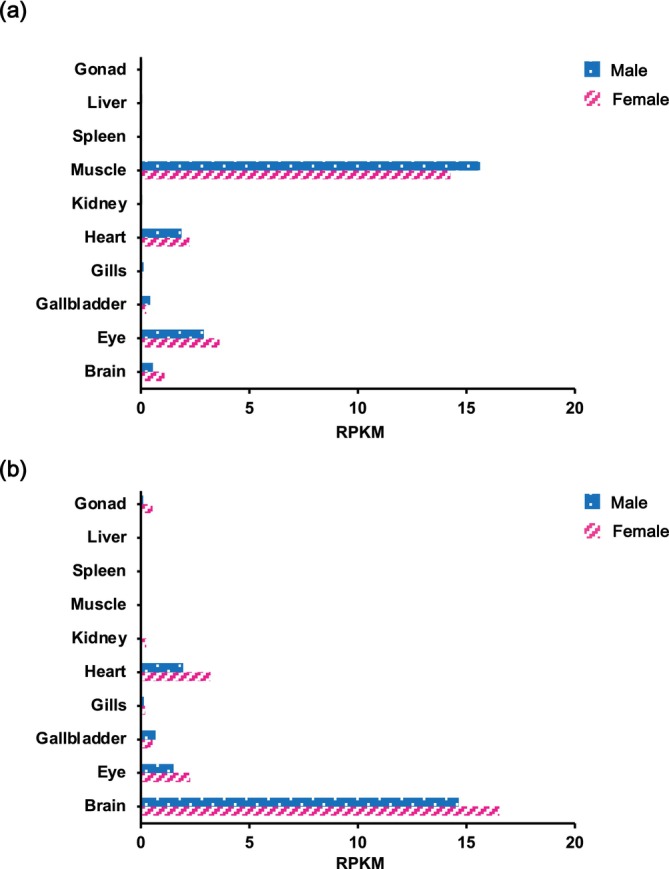
RNA sequencing‐derived expression profiles of *nt5c1aa* (a) and *nt5c1ab* (b) in different organs of male and female Cab strain medaka. Total RNA from each organ was isolated from pooled samples of 8–16 individuals to minimize variation among fish. Transcript abundance was calculated as reads per kilobase of exon per million mapped reads (RPKM).

To clone *nt5c1a* from the Japanese medaka Cab strain, we searched the NCBI genome database and identified two types of *nt5c1a* in the Japanese medaka Hd‐rR strain as human *nt5c1a* orthologs. Thus, we termed these two *nt5c1a* genes *nt5c1aa* (Hd‐rR: GenBank accession number LOC101170199) and *nt5c1ab* (Hd‐rR: GenBank accession number LOC101156450). RNA sequencing (RNA‐seq) analysis of the Cab strain showed that the expression levels of *nt5c1aa* and *nt5c1ab* were the highest in the muscles and brains of males, respectively (Figure 2a,b), revealing distinct tissue‐specific expression patterns for the two paralogs. Using cDNA from the muscle and brain tissues, the coding sequences (CDSs) of *nt5c1aa* or *nt5c1ab* were isolated using reverse transcription–polymerase chain reaction (RT‐PCR) and then cloned into each plasmid with reporter genes (Figure S1c). The full‐length open reading frames of Cab *nt5c1aa* and *nt5c1ab* cDNA were comprised of 1260 bp encoding 420 amino acid (AA) residues and 1110 bp encoding 370 AA residues, respectively. The nucleotide sequences of *nt5c1aa* and *nt5c1ab* in the medaka Cab strain mostly corresponded to those of the Hd‐rR strain, but several mutations were detected. Six silent mutations and two missense mutations were identified in the nucleotide sequence of *nt5c1aa*, whereas six silent mutations and one missense mutation were found in *nt5c1ab* (Figure S3a,b).

**FIGURE 3:**
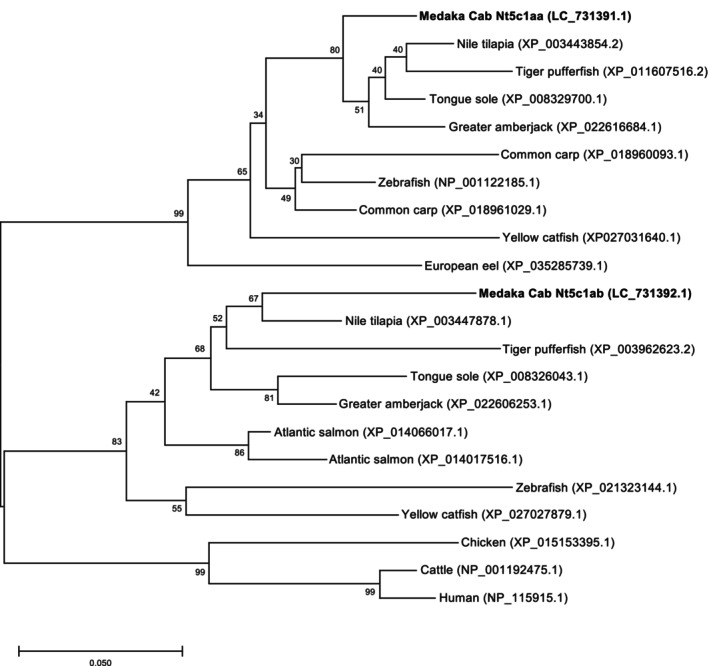
Phylogenetic analysis of Nt5c1aa and Nt5c1ab proteins. The phylogenetic tree shows the evolutionary relationships of the medaka *Oryzias latipes* Nt5c1aa (LC_731391.1) and Nt5c1ab (LC_731392.1) proteins with their homologs from various other vertebrate species. Species names and their corresponding accession numbers are indicated. The numbers at the nodes represent bootstrap values (%), obtained from 1000 replicates. The scale bar at the bottom indicates the genetic distance of 0.050 amino acid substitutions per site.

**FIGURE 4:**
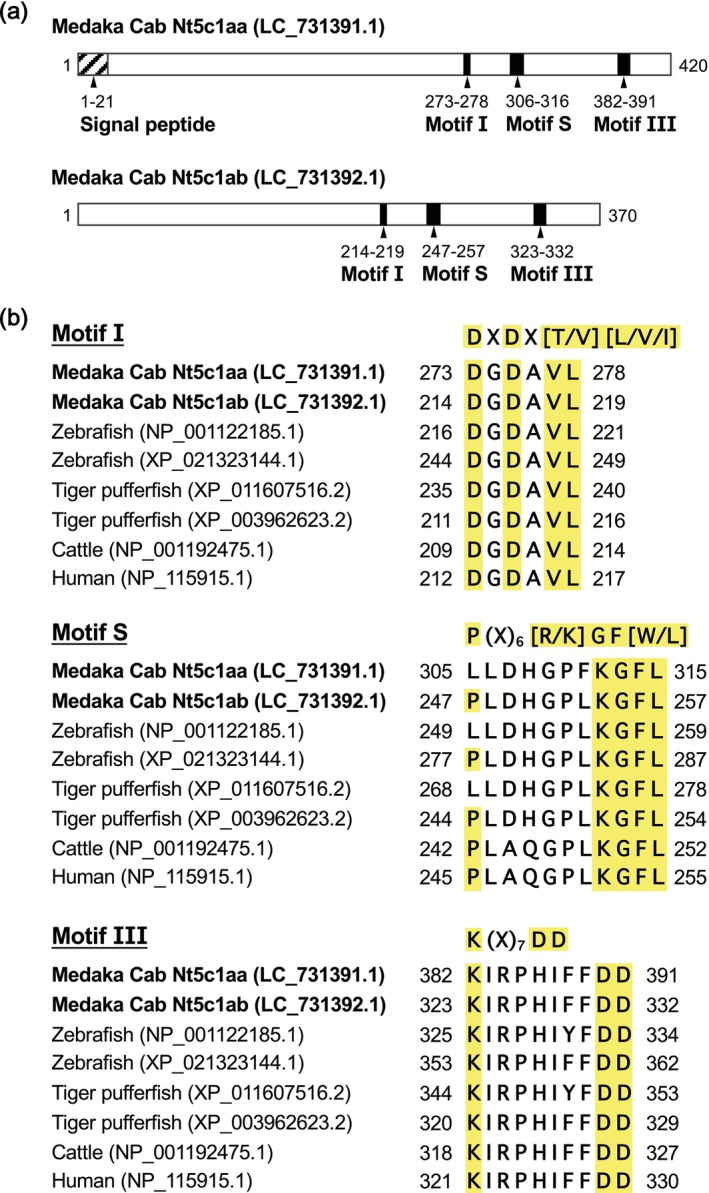
Comparison of medaka Nt5c1aa and Nt5c1ab protein structures. (a) Schematic diagrams of the medaka *Oryzias latipes* Nt5c1aa (LC_731391.1) and Nt5c1ab (LC_731392.1) protein structures. The numbers represent amino acid positions. Key functional domains, including the N‐terminal signal peptide and conserved motifs (Motif I, S, and III), are indicated. (b) Sequence alignment of the highly conserved motifs (I, S, and III) from medaka and other representative species (zebrafish, tiger pufferfish, cattle, and humans). The yellow‐highlighted sequences indicate the consensus sequences shared among all the listed species. Amino acid positions are numbered relative to each full‐length protein sequence.

To examine the evolutionary background of these two paralogs identified in medaka, we conducted a molecular phylogenetic analysis comprising major aquaculture fish species, humans, and other vertebrates. As shown in Figure 3, phylogenetic analysis revealed that all fish Nt5c1a sequences formed a cluster separate from those of mammalian Nt5c1a. Within the fish clade, Cab Nt5c1aa and Nt5c1ab were clustered together with several other Nt5c1a sequences from Perciformes fish, such as greater amberjack ( *Seriola dumerili* ) and Nile tilapia ( *Oreochromis niloticus* ). The phylogenetic tree also showed that Nt5c1aa and Nt5c1ab belong to different sub‐clades, highlighting the distinct evolutionary trajectories of the two paralogs identified in this study. To predict the functions of the two paralogs, we performed an in silico analysis of their sequence features. Our in silico analysis, conducted using SignalP, identified the possible secretion signal peptides in the first 28 AAs (^1^MLQTAAALSVYLVGGFFFVLG^21^) of Nt5c1aa, but not in Nt5c1ab (Figure S4a,b). A previous study reported that Nt5c1a possesses several characteristic motifs involved in enzyme activity (Hunsucker et al. 2005). Specifically, two motifs involved in catalysis (motifs I and III) and one motif involved in substrate recognition (motif S) have been identified in Nt5c1a in other vertebrates, such as humans. We therefore evaluated whether these motifs are conserved across species in medaka Nt5c1aa and Nt5c1ab. As a result, the amino acid sequence of Cab Nt5c1aa contained motif I, DXDX[T/V][L/V/I], motif III, K(X)_7_DD, and a slightly mutated motif S, P(X)_7–8_[R/K]GF[W/L], in which the first amino acid had been substituted from proline to leucine (P → L), whereas that of Cab Nt5c1ab contained fully conserved motif I, III, and S (Figure 4a,b). Collectively, these analyses suggest that the medaka *nt5c1a* gene has diverged into two paralogs with different structural characteristics. While *nt5c1ab* retains the conserved catalytic motifs necessary for IMP degradation, *nt5c1aa* appears to have lost these features. Therefore, in a subsequent experiment, we compared and evaluated the IMP‐degrading activity of both paralogs.

### 2.3 Evaluation of the IMP‐Degrading Activity of nt5c1a Paralogs Using the Optimized Transient Assay System

**FIGURE 5:**
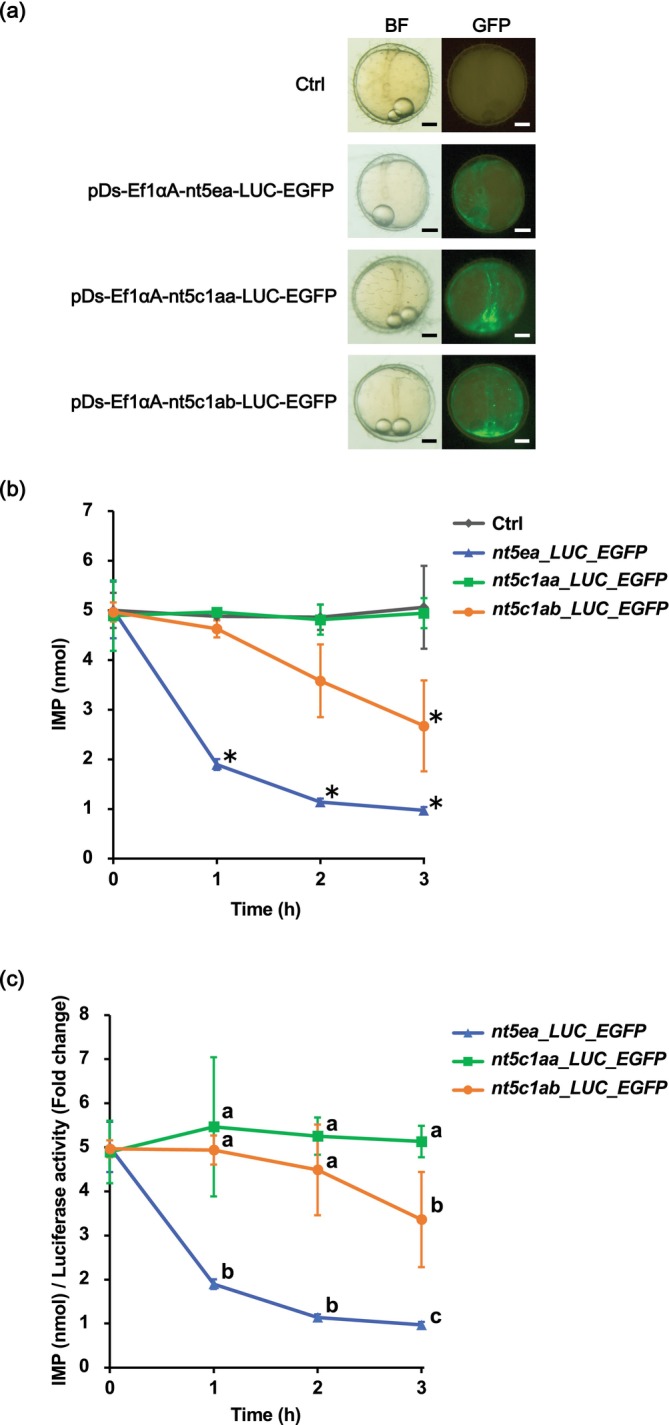
Functional analysis of medaka Nt5 family members. (a) Representative fluorescence images of embryos at 1 day post‐fertilization. Green fluorescence was detected from embryos injected into the cytoplasm with pDs‐Ef1aA‐LUC‐EGFP, pDs‐Ef1aA‐nt5c1aa‐LUC‐EGFP, or pDs‐Ef1αA‐nt5c1ab‐LUC‐EGFP constructs. Embryos without microinjection were used as a control (Ctrl). Except for in the Ctrl, green fluorescence was observed in the embryos. BF, bright field; GFP, green fluorescent protein. Scale bars = 0.2 μm. (b) IMP degradation activity of Nt5 family members in medaka. Data are presented as the mean ± SD of three measurements from different enzyme preparations. The asterisks indicate that the values are significantly different from those of the Ctrl group according to one‐way ANOVA followed by Tukey's HSD test (*p < 0.05). (c) Comparison of the IMP‐degrading activities among Nt5 family members after normalization with LUC luminescence. Different letters indicate significant differences among the groups at each time point according to one‐way ANOVA followed by Tukey's HSD test.

We used the optimized expression system to assay the IMP‐degrading activities of Cab Nt5c1aa, Nt5c1ab, and Nt5ea. Cab Nt5ea possesses IMP‐degrading activity (Murakami et al. 2022), therefore we used it as a reference to compare the IMP‐degrading activities of other enzymes. The three plasmids containing the *ef1αA* promoter (Figure S1c) were injected into the yolk, which induced green fluorescence in the embryos at 1 dpf (Figure 5a). The IMP‐degrading activities were examined by mixing the embryo extracts with IMP in the reaction solution. The amount of IMP in the group injected with pDs‐Ef1αA‐nt5c1ab‐LUC‐EGFP gradually decreased from the start of the reaction and was significantly lower than that in the control (uninjected) group at 3 h after the reaction (Figure 5b). This confirms that Nt5c1ab possesses functional IMP‐degrading activity. The group injected with pDs‐Ef1αA‐nt5ea‐LUC‐EGFP showed the most rapid reduction in IMP concentration, with nearly complete degradation occurring by 3 h, and the amount of IMP was significantly different from that of the control group between 1 and 3 h after reaction. Previously, we demonstrated that the fusion protein LUC‐EGFP is not involved in IMP degradation (Murakami et al. 2022); these results indicated that Nt5c1ab and Nt5ea degrade IMP. However, the group injected with pDs‐Ef1αA‐nt5c1aa‐LUC‐EGFP showed mostly constant IMP values, consistent with those of the control group. This result suggests that Nt5c1aa does not have IMP‐degrading activity.

To accurately compare the IMP‐degrading activities of Nt5c1aa, Nt5c1ab, and Nt5ea, we normalized the amount of IMP to the value of LUC luminescence (Figure S5). This normalization accounts for potential variations in the expression levels of the injected constructs among individual embryos. As shown in Figure 5c, LUC‐normalized IMP levels decreased rapidly in embryos expressing Nt5ea, whereas a more gradual decrease was observed in embryos expressing Nt5c1ab. In contrast, embryos expressing Nt5c1aa showed little change in IMP levels over the reaction period. These results showed that, among the Nt5 proteins tested, Nt5ea has the strongest IMP‐degrading activity, Nt5c1ab has intermediate IMP‐degrading activity, and Nt5c1aa has no IMP‐degrading activity.

To further validate the normalization strategy based on LUC luminescence, we additionally normalized IMP levels to total protein content (Figure S6a). Total protein levels did not differ significantly among embryos expressing Nt5ea, Nt5c1aa, or Nt5c1ab fused with LUC‐EGFP (Figure S6b). Consistent with the LUC‐normalized analysis, total protein‐normalized IMP levels decreased rapidly in Nt5ea‐expressing embryos, declined more gradually in Nt5c1ab‐expressing embryos, and remained largely unchanged in Nt5c1aa‐expressing embryos. These results indicate that normalization using LUC activity provides a reliable proxy for enzyme expression levels and does not bias the comparative evaluation of IMP‐degrading activities among Nt5 proteins.

To further quantify the differences in IMP‐degrading activities, initial reaction rates were calculated based on the decrease in LUC‐normalized IMP levels during the early phase of the reaction (0–2 h). Nt5ea exhibited the highest reaction rate, which was significantly greater than those of Nt5c1aa and Nt5c1ab (Table S1). In contrast, Nt5c1aa showed no measurable reaction rate, whereas Nt5c1ab displayed a modest but consistent IMP‐degrading activity. Overall, the quantitative assessment confirms that Nt5c1ab retains its catalytic function while Nt5c1aa has lost its IMP‐degrading activity, providing a functional basis for further investigating the molecular mechanisms underlying their divergence.

### 2.4 Structural Insights Into Nt5c1aa and Functional Rescue by Site‐Directed Mutagenesis

The lack of IMP‐degrading activity detectable in Nt5c1aa may be due to the point mutation (P → L) present in motif S. To investigate the structural impact of the mutation on Nt5c1aa function, we performed a structural analysis using AlphaFold2. Predicted structures for both the wild‐type and mutant (L305P) Nt5c1aa proteins showed high local confidence scores (predicted local distance difference test; pLDDT > 90) in most structured regions, indicating reliable models (Figure S7a,b). The regions with lower confidence scores (cyan to orange) were primarily found in the flexible loop areas. Structural superposition of the wild‐type and mutant models revealed that the L305P substitution induced a local conformational change in motif S without affecting the whole protein architecture (Figure S7c). Notably, the side chain orientation at residue 305 differed substantially between the two models, potentially influencing substrate recognition or positioning (Figure S7d).

**FIGURE 6:**
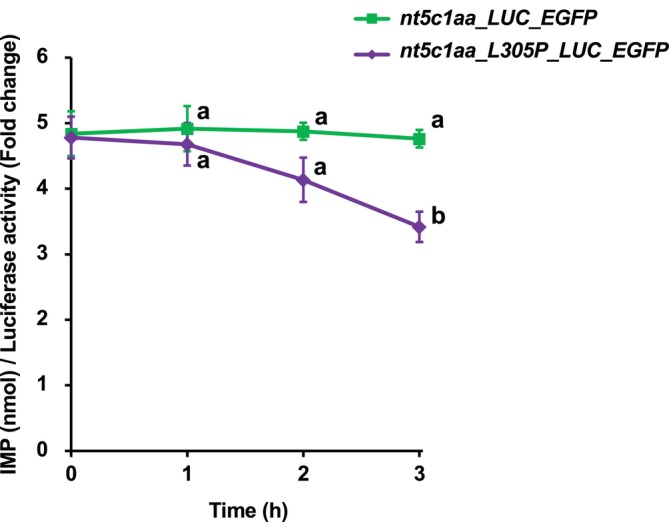
Luciferase‐normalized time‐course analysis of IMP degradation in embryos expressing wild‐type Nt5c1aa or the L305P mutant. Time‐course analysis of IMP levels normalized to luciferase activity in embryos expressing Nt5c1aa‐LUC‐EGFP or Nt5c1aa‐L305P‐LUC‐EGFP. Normalization to LUC luminescence was performed to account for potential differences in expression levels of the fusion proteins among embryos. Nt5c1aa‐L305P‐expressing group showed significantly lower IMP levels at 3 h compared with Nt5c1aa‐expressing group, whereas no significant differences were detected at earlier time points (0–2 h). These results indicate that L305P substitution confers a modest enhancement of IMP‐degrading activity that becomes apparent during later stages of the reaction. Data are presented as mean ± SD. Different letters indicate significant differences among the groups at each time point according to one‐way ANOVA followed by Tukey's HSD test.

These structural predictions prompted us to examine whether the L305P substitution affects enzymatic function. Comparison of IMP degradation activities showed that the initial reaction rates did not differ significantly between Nt5c1aa (0.02 ± 0.14) and Nt5c1aa‐L305P (−0.32 ± 0.33) (Table S2). Time‐course analysis of IMP levels showed that embryos expressing Nt5c1aa‐L305P exhibited significantly lower IMP levels than those expressing Nt5c1aa at 3 h, whereas no significant differences were observed at earlier time points (Figure S8a). Normalization of IMP values by LUC luminescence yielded nearly identical temporal patterns (Figure 6, Figure S8b), indicating that the observed difference was not attributable to an artifact of normalization. These results indicate that the L305P substitution confers a modest but measurable enhancement of IMP‐degrading activity without substantially altering the early reaction kinetics.

## 3 Discussion

Despite clear differences in LUC activity, the lack of significant differences in EGFP‐positive rates highlights the distinct nature of the two reporter assays. In this study, EGFP expression was evaluated qualitatively by visual inspection, in which embryos exhibiting even faint or localized fluorescence were classified as positive. While this approach is suitable for confirming successful expression, it has limited sensitivity for distinguishing differences in overall expression levels among experimental conditions. In contrast, LUC activity provides a quantitative measure of total reporter protein within the embryo. Thus, we employed LUC‐based assays to compare the efficiency of transient expression systems.

We revealed that the combination of a plasmid containing the *ef1αA* promoter with yolk injection induced the highest protein expression levels at 1 dpf. This approach has two major advantages in simplifying the production of exogenous proteins. First, yolk injection enhances throughput by eliminating the need for precise egg orientation. Unlike cytoplasmic injection, which requires time‐consuming manipulation to align the cytoplasm with the needle (Murakami and Kinoshita 2018), yolk injection enables high‐throughput processing of embryos. Second, this method reduces the labor required for preparation of the injectant. While mRNA‐based assays necessitate in vitro synthesis for every target gene, the DNA‐based method utilizes plasmids directly. Although the *Ac*/*Ds* transposon system requires *Ac* mRNA (Boon Ng and Gong 2011), a single batch of *Ac* mRNA can be synthesized, cryopreserved, and used for any target gene. Thus, the DNA‐based method streamlines the workflow from preparation to injection, potentially accelerating functional genomic studies in fish.

Of the six injection combinations tested in this study, the injection of the plasmid containing the *ef1αA* promoter into the yolk was found to yield the highest levels of transient expression. Although the exact reason this combination was superior remains to be fully elucidated, it may be due to the high transcriptional and translational activities of extraembryonic cells. Importantly, because the yolk itself lacks nuclei and the molecular machinery required for transcription, it is unlikely that the yolk mass directly produced the reporter proteins. Medaka have a single yolk mass in the egg; therefore, the molecules injected into the yolk struggle to pass through the yolk‐cytoplasm boundary and may remain in the yolk to serve as the substrate for expression (Goto et al. 2019). While direct experimental evidence was not obtained in this study, we propose that the yolk syncytial layer (YSL) is a suitable site for the expression of foreign proteins. The YSL is a transient extraembryonic syncytial structure that forms at the early blastula stage and persists through larval stages (Concha and Reig 2022). In zebrafish, the YSL and blastoderm extend down toward the vegetal pole to cover and enclose the yolk by 10 h post‐fertilization during epiboly (Carvalho and Heisenberg 2010). Furthermore, the YSL of some species is known to contain giant polyploid nuclei, and that in zebrafish has high enough transcriptional and translational activity to express foreign genes. Based on these characteristics, it is possible that reporter proteins synthesized in the YSL diffused along the yolk surface, causing the observed broad fluorescence. However, as we did not perform co‐localization analysis with YSL‐specific markers, this remains a speculative explanation. Further detailed studies, such as imaging using YSL‐specific markers or spatial transcriptomics, are needed to determine the exact location of expression and to confirm the functional involvement of the YSL in this system.

Several studies have reported the use of fish embryos as expression systems. For example, goldfish luteinizing hormone has been generated using rainbow trout embryos at four dpf as bioreactors (Morita et al. 2004). Moreover, we previously established a medaka‐embryo bioreactor system in which foreign proteins were delivered from the liver and accumulated in eggs via a vitellogenin‐derived signal sequence (Murakami et al. 2019). However, the expression system developed in the present study offers several advantages over these systems. The novel system was optimized for rapid and efficient expression in early medaka embryos, achieving high expression levels as early as 1 dpf. This feature facilitates functional gene screening in fish embryos and enables in vivo evaluation of gene function. While these advantages exist, the trade‐off between expression efficiency and embryo viability must also be considered. Our results showed that yolk injection, despite its superior reporter activity, significantly reduced the survival rate compared to cytoplasmic injection. This reduction in viability might be due to the physical stress on the yolk membrane or the disruption of the yolk nutrient environment during the microinjection process. Unlike large‐scale bioreactor methods, this system is not designed for mass recombinant protein production. Its transient expression period of 1–3 days post‐injection may be shorter than that of systems that rely on maternal accumulation or long‐term embryo culture. Consequently, evaluating genes requiring later developmental stages may prove challenging. Furthermore, due to the transient nature of expression, unlike some transgenic approaches aimed at long‐term expression, transmission to the germline and stable integration are not addressed. Overall, this system specializes in functional gene screening in fish and is intended to complement, rather than replace, large‐scale production‐oriented egg or embryo expression platforms.

Nt5c1a has been annotated as a cytosolic 5′‐nucleotidase in several vertebrates (Skladanowski and Newby 1990; Garvey et al. 1998; Hunsucker et al. 2001), but its enzymatic degradation activity toward IMP has not been experimentally verified in fish. The present study provides novel experimental evidence that Nt5c1a in fish functions as an IMP‐degrading enzyme. Given this finding, it is important to consider the evolutionary context of *nt5c1a* genes in teleosts. Teleost fish have undergone an additional round of whole‐genome duplication compared with mammals, resulting in the retention of multiple paralogous gene copies (Glasauer and Neuhauss 2014). Consistent with this evolutionary background, two *nt5c1a* paralogs, *nt5c1aa* and *nt5c1ab*, are present in medaka. RNA‐seq data indicated that *nt5c1aa* is predominantly expressed in the muscles, whereas *nt5c1ab* is highly expressed in the brain. Using the optimized expression system established in this study, we demonstrated that *nt5c1ab* exhibited IMP‐degrading activity, while *nt5c1aa* did not. Considering their distinct expression patterns and enzymatic properties, it is plausible that *nt5c1aa* has undergone sub‐functionalization during teleost evolution (Glasauer and Neuhauss 2014). Our phylogenetic analysis further indicates that many species used in aquaculture, such as Nile tilapia, possess two *nt5c1a* paralogs that form two distinct clusters. Consequently, genes within the same cluster likely share evolutionary origins and may possess comparable biochemical or physiological functions.

The lack of detectable IMP‐degrading activity by Nt5c1aa may be due to amino acid substitutions in key substrate recognition motifs. Indeed, sequence alignment revealed that Nt5c1aa carries a substitution of proline to leucine (P → L) within the conserved substrate‐recognition motif P(X)_7–8_[R/K]GF[W/L], which has been identified as critical for catalytic function in mammalian Nt5c1a (Hunsucker et al. 2005). Although Nt5c1ab exhibited measurable IMP‐degrading activity in our expression system, its activity appeared weaker than that of Nt5e. Furthermore, normalization of IMP levels to total protein content yielded results comparable to those obtained using LUC activity, suggesting that the observed differences in IMP degradation are not driven by variation in overall protein abundance.

Notably, Nt5e and Nt5c1ab are unlikely to function redundantly on the same IMP pool because they are spatially segregated within the cell. We previously demonstrated that the disruption of *nt5e* function effectively suppressed IMP degradation in postmortem muscle, thereby enhancing IMP retention (Murakami et al. 2022). Nt5e acts as an extracellular Nt5 at the cell surface and primarily regulates extracellular purine metabolism, whereas Nt5c1ab is a cytosolic enzyme that processes intracellular nucleotides. Therefore, the pronounced IMP retention observed in *nt5e*‐deficient fish is most likely attributable to the loss of the dominant extracellular IMP‐degrading pathway and cannot be fully compensated by cytosolic Nt5c1ab. Considering that Nt5c1ab exhibits IMP degradation activity, albeit at a lower level than Nt5e, a compound knockout of *nt5c1ab* alone or together with *nt5e* could further contribute to maintaining higher IMP levels in fish muscle.

The present structural and functional analyses together provide mechanistic insight into the loss of IMP‐degrading activity in Nt5c1aa. AlphaFold2 predictions indicated that the L305P substitution does not disrupt the overall fold of the protein but instead induces a localized conformational alteration within motif S, a region implicated in substrate recognition. This observation suggests that the catalytic deficiency of Nt5c1aa is unlikely to result from global misfolding or instability, but rather from subtle structural perturbations affecting substrate interaction.

Functional assays of the L305P mutant further support this interpretation. Although the substitution did not significantly alter initial reaction rates, embryos expressing Nt5c1aa‐L305P showed reduced IMP levels at later time points compared with wild‐type Nt5c1aa, indicating a modest restoration of enzymatic activity. Importantly, the partial rather than complete functional rescue observed here suggests that additional residues or structural elements may also participate in substrate binding or catalysis. Future mutational and biochemical analyses are therefore required to fully define the structural determinants governing Nt5c1a enzymatic specificity in teleosts.

These findings indicate that *nt5c1a* may be a promising molecular target for meat‐quality improvement in both fish and other livestock species. In support of this, studies in chickens have reported that *nt5c1a* expression varies depending on rearing conditions, which may be associated with differences in IMP content (Zhang et al. 2018). A more comprehensive understanding of the IMP metabolic network, including *nt5c1a* and *nt5e* activity, across different animal species will be crucial for advancing molecular breeding strategies aimed at enhancing umami‐related compounds. The insights obtained from medaka in this study are expected to provide a genetic foundation for future efforts to enhance meat quality through targeted genome modification.

## 4 Methods

### 4.1 Fish

Closed colonies of the Cab strain were obtained from Dr. Todo's group at the Radiation Biology Center, Kyoto University, Kyoto, Japan. Fish were maintained in an aquarium at 28°C under a 14/10‐h light/dark cycle. Fish use and care in this study were conducted in strict accordance with the Guidelines for the Care and Use of Research Animals adopted by the Kindai University Committee on Animal Research and Bioethics (KAAG‐2021‐014). All efforts were made to minimize animal suffering.

### 4.2 Construction of Plasmids to Optimize the Transient Assay

To optimize the transient expression system, two plasmids carrying different promoters were constructed. Each promoter drove the expression of a dual reporter gene cassette encoding firefly *LUC* and *EGFP*. Insert fragments were amplified using PCR with PrimeStar GXL DNA Polymerase (TaKaRa) under the following protocol: 30 cycles of 98°C for 10 s, 55°C for 10 s and 68°C for 30 s. Both the PCR‐amplified inserts and the plasmid backbones were digested with appropriate restriction enzymes and ligated using a Ligation High Ver.2 Kit (TOYOBO) to generate the desired constructs. Plasmids were extracted using the Wizard Plus SV Minipreps DNA Purification System Kit (Promega) following the manufacturer's instructions and verified using Sanger sequencing. The primer sequences and plasmid maps used in this study are provided in Table S3 and Figure S1a,b.

### 4.3 Preparation of Plasmids for RNA Synthesis and Microinjection

To eliminate residual RNase activity in the extracted plasmid solutions, four plasmids, including pDs‐Actb‐LUC‐EGFP, pDs‐Ef1αA‐LUC‐EGFP, pAcII (Inoue et al. 2016), and pCS2‐LUC‐EGFP‐pA (Murakami et al. 2022), in 50 μL of 5 mM Tris–HCl buffer (pH 8.5) were incubated with 5 μL of 10% sodium dodecyl sulfate (SDS) and 2 μL of Proteinase K (20 mg/mL) at 55°C for 30 min. The incubated solutions were purified with the NucleoSpin Gel and PCR Clean‐up kit using the Buffer NTB (Macherey‐Nagel).

For in vitro transcription of *Ac* and *LUC*‐*EGFP* mRNA, the plasmids of pAcII and pCS2‐LUC‐EGFP‐pA were linearized using BamHI and NotI, respectively, and purified as described above. The plasmids were used as a template to synthesize capped mRNAs using the Message mMachine SP6Kit (Life Technologies) as described previously (Ansai and Kinoshita 2014). All synthesized mRNAs were purified using the RNeasy Plus Mini Kit (Qiagen) to eliminate the template DNA without DNase treatment for microinjection.

### 4.4 Microinjection

Microinjection was performed under an upright microscope XF‐PH‐21 (Nikon) equipped with a micromanipulator M‐152 (Narishige) and a microinjector IM‐6 (Narishige). Embryos were placed in a silicone plate with wells to securely hold the medaka embryos (Murakami and Kinoshita 2018). To establish an efficient expression system, mRNA‐ and DNA‐based approaches were tested. For the mRNA‐based approach, *LUC‐EGFP* mRNA was prepared at 100 ng/μL in nuclease‐free water. For the DNA‐based approach, a mixture containing 10 ng/μL of pDs‐actb‐LUC‐EGFP or pDs‐Ef1αA‐LUC‐EGFP plasmid DNA and 100 ng/μL of *Ac* mRNA was prepared. Approximately 2–4 nL of each mixture was injected into the cytoplasm or yolk of each embryo at the one‐cell stage (Kinoshita et al. 2000). The plasmids with the *Ds* sequences combined with *Ac* mRNA were introduced into the embryos. The *Ac* transposase recognizes the inverted repeats flanking the *Ds* element, leading to the excision and insertion of the *Ds* sequence into the host genome (Boon Ng and Gong 2011; Lazarow et al. 2013). After injection, the embryos were incubated at 28°C until further analysis.

### 4.5 Microscopic Observation of Fluorescence

Embryos injected with each mixture were observed using a fluorescence stereomicroscope SZX16 (Evident) with SZX2‐FGFP (for GFP) filter set. Microscopic images were captured using a DP73 camera and cellSens image acquisition software (Evident).

### 4.6 Reporter Assay for the Detection of LUC Activity

Bioluminescence was detected in the embryos injected with each mixture to evaluate the expression efficiency of each method. For each experimental group, twelve embryos exhibiting green fluorescence were used for luminescence analysis. The embryos were divided into four independent sets, each consisting of three embryo pools. Each set of embryos was placed in a 1.5‐mL microtube containing 150 μL of reporter lysis buffer (Promega) and homogenized with a pestle. After freezing (−80°C) and thawing (room temperature) to ensure complete lysis, 100 μL of the supernatant from each lysate was transferred into the wells of a 96‐well microplate. The luminescent reaction was initiated by adding 10 μL of prepared luciferase assay reagent II using assay buffer (Promega) to each well. Bioluminescence intensity was measured using a GloMax 96 microplate luminometer (Promega). The measured bioluminescence intensity was normalized to the concentration of total protein extracted from each lysate and expressed as RLU. The protein concentrations in 4 μL of the same lysate were determined using the Bradford Protein Assay Kit (TaKaRa) according to the manufacturer's protocol. All procedures were independently repeated three times to ensure reliability and reproducibility.

### 4.7 Plasmid DNA Dose–Response Analysis

To evaluate the effect of plasmid concentration on embryo survival and transgene expression, embryos were injected with pDs‐Ef1αA‐LUC‐EGFP plasmid at final concentrations of 1, 10, or 20 ng/μL into the yolk. Survival rates were assessed at 1 dpf. LUC activity was measured as described above, and values were normalized and expressed as fold change relative to the 1 ng/μL group.

### 4.8 Identification and Cloning of nt5c1aa and nt5c1ab in Medaka

The NCBI genome database of the medaka Hd‐rR strain (GenBank accession number ASM223467v1) was screened using the human *nt5c1a* nucleotide sequence (GenBank accession number 84618) as a query. The transcription levels of the *nt5c1a* genes were examined using our previously obtained RNA‐seq data (DDBJ accession number DRA014727) (Murakami et al. 2020). The expression profile was constructed using the total RNA of 10 different tissues from Cab males and females, which helped to select the optimal tissues and sex for subsequent RNA extraction. The CDSs of the Cab Nt5c1aa and Nt5c1ab were isolated using RT‐PCR. Total RNA was extracted from the male muscle or brain of the Cab strain using an RNeasy Plus Mini kit (Qiagen). One microgram of total RNA was used to synthesize first‐strand cDNA with random hexamers and SuperScript III Reverse Transcriptase (Thermo Fisher Scientific) according to the manufacturer's instructions. The cDNA was used for PCR amplification of the CDSs of *nt5c1aa* or *nt5c1ab* with PrimeStar GXL DNA Polymerase (TaKaRa) using the primers Insert‐nt5c1aa‐FW/Insert‐nt5c1aa‐RV or Insert‐nt5c1ab‐FW/Insert‐nt5c1ab‐RV (Table S3). The PCR conditions were as follows: incubation at 94°C for 2 min, followed by 35 cycles at 98°C for 10 s, 58°C for 30 s, and 68°C for 50 s. The PCR products collected in the agarose gels were purified using a NucleoSpin Gel and PCR Clean‐up kit (Macherey‐Nagel). The amplicons of *nt5c1aa* and *nt5c1ab* were cloned to construct the plasmids pDs‐Ef1αA‐nt5c1aa‐LUC‐EGFP and pDs‐Ef1αA‐nt5c1ab‐LUC‐EGFP (Figure S1c) using In‐Fusion HD Cloning Kit (Takara) following the manufacturer's instructions. To evaluate the IMP‐degrading activities of Nt5c1aa and Nt5c1ab, pDs‐Ef1αA‐LUC‐EGFP was used as the control plasmid (Figure S1b). The plasmid pDs‐Ef1αA‐nt5ea‐LUC‐EGFP (Figure S1c), which contained *nt5ea* that was previously analyzed (Murakami et al. 2022), was constructed using a PCR‐based method with the primer Insert‐nt5ea‐FW/Insert‐nt5ea‐RV (Table S3). The methods for constructing these plasmids are described in Figure S1a–c. Plasmids were extracted using the Wizard Plus SV Minipreps DNA Purification System Kit (Promega) following the manufacturer's instructions and were sequenced using the Sanger method.

### 4.9 In Silico Analysis of Nt5c1aa and Nt5c1ab

Alignment of the AA and nucleotide sequences of Nt5c1aa and Nt5c1ab was performed using Clustal Omega (http://www.ebi.ac.uk/Tools/msa/clustalo/). The CDSs of *nt5c1a* from teleost and non‐teleost vertebrates ( *Homo sapiens* , *Bos taurus* , and *Gallus gallus* ) were collected using ORTHOSCOPE v1.5.1 (http://yurai.aori.u‐tokyo.ac.jp/orthoscope/Vertebrata.html) and BLAST searches (https://blast.ncbi.nlm.nih.gov/Blast.cgi). Phylogenetic analysis was performed using the neighbor‐joining method in the MEGA X program, and bootstrap sampling was conducted with 1000 repetitions.

Structural models of wild‐type and mutant Nt5c1aa proteins were generated using AlphaFold2 v1.5.5 (https://colab.research.google.com/github/sokrypton/ColabFold/blob/main/AlphaFold2.ipynb). Amino acid sequences were provided as input, and the top‐ranked predicted models were used for subsequent analyses. Model confidence was evaluated using the pLDDT scores provided by AlphaFold2. Structures were visualized and analyzed using PyMOL v3.1.6.1 (https://www.pymol.org/). Structural superposition of wild‐type and mutant models was performed to assess local conformational differences, with particular focus on motif S (residues 305–315) and residue 305.

### 4.10 Evaluation of the IMP‐Degrading Activity of Wild‐Type and Mutant Nt5 Proteins

Four plasmids, pDs‐Ef1αA‐nt5ea‐LUC‐EGFP, pDs‐Ef1αA‐nt5c1aa‐LUC‐EGFP, pDs‐Ef1αA‐nt5c1aa‐L305P‐LUC‐EGFP, and pDs‐Ef1αA‐nt5c1ab‐LUC‐EGFP, at 10 ng/μL were injected into the yolk of medaka embryos with 100 ng/μL *Ac* mRNA (Figure S1c,d). Embryos expressing the reporter fusion (*LUC‐EGFP*) and *nt5* family members (*nt5ea*, *nt5c1aa*, the *nt5c1aa* L305P mutant, or *nt5c1ab*) were collected at 1 dpf. Uninjected eggs were used as the controls. A total of 40 embryos that exhibited green fluorescence were selected from each group and pooled into 50 μL of phosphate‐buffered saline (PBS; pH 7.4) in a 1.5 mL microtube and homogenized using a motorized pestle. After centrifugation at 15,000 *g* for 10 min at 4°C, the supernatant was divided into two portions: one for LUC luminescence measurement (to normalize expression level) and another for the enzymatic assay. LUC activity was measured using a GloMax 96 microplate luminometer according to the method described in the above section. For the IMP‐degradation assay, 25 μL of the supernatant was added to a reaction mixture containing 50 mM Tris–HCl (pH 7.2) and 5 mM MgCl_2_ with a final volume of 50 μL. Each mixture was preincubated for 10 min at 28°C, which represents the standard physiological and rearing temperature for medaka. This temperature was selected to ensure the assay reflects the enzymatic characteristics within a teleost‐specific cellular environment, rather than the 37°C typically used for mammalian studies. The reaction was initiated by adding the substrate, IMP, to a final concentration of 100 μM and was stopped by adding 100 μL of 10% perchloric acid. Samples were neutralized on ice with 1 N KOH, centrifuged at 15,000 *g* for 10 min at 4°C, and filtered through a 0.45 μm membrane. The supernatants were analyzed by HPLC using a column (TSK gel ODS 80Ts, 4.6 × 250 mm). The HPLC analysis was performed based on Kinoshita et al. (2013), with the detailed conditions described below. The mobile phase consisted of solvent A (0.1 M NaH_2_PO_4_, pH 4.1) and solvent B (acetonitrile) at a flow rate of 1.0 mL/min. The gradient program was as follows: 2% B from 0 to 5 min, increased linearly to 10% B from 5 to 15 min, held at 10% B until 25 min, returned to 2% B at 25.1 min, and equilibrated at 2% B until 40 min. The column was washed with 100% B and re‐equilibrated with 2% B prior to the next run. The column temperature was maintained at 30°C, IMP was detected at 254 nm, and the injection volume was 10 μL. IMP remaining in the reaction was quantified, and activity was calculated relative to LUC luminescence or total protein. Total protein concentrations were measured using the Bradford Protein Assay Kit (TaKaRa) as described above. Reaction rates were calculated from the decrease in IMP levels during the initial linear phase of the reaction (0–2 h). For each experimental group, IMP concentrations were plotted against reaction time, and the reaction rate was determined as the slope of the linear regression. Analyses were performed independently for each experimental replicate.

### 4.11 Statistical Analysis

All statistical analyses were performed using R version 4.2.2 (http://www.r‐project.org). Quantitative data were compared using one‐way ANOVA followed by Tukey's HSD test. Survival rates, calculated as the number of surviving embryos relative to the number of injected embryos, were analyzed using Fisher's exact test. Statistical comparison of LUC activity between the two groups was performed using Welch's *t*‐test. Differences were considered significant at *p* < 0.05.

## Author Contributions

Y.M. designed and conducted the experiments and wrote the manuscript. T.H. assisted with luminescence quantification. M.A. contributed to the chemical analysis for IMP quantification. T.K. provided guidance on data interpretation and the overall project direction. All authors read and approved the final manuscript.

## Funding

This study was supported by Japan Society for the Promotion of Science (20J00697, 22K14940, and 25K18286) and Japan Science and Technology Agency (JPMJPF2114) (Y. M.).

## Supporting information


**Figure S1:** Schematic diagram of the plasmids used. To induce transposon‐mediated high expression in medaka embryos, plasmids with different promoters were constructed using a two‐step process (a, b). (a) Construction of the plasmid containing the reporter gene. To obtain a backbone fragment containing 5′‐and 3′‐*Ds* elements and an SV40 polyA signal (*pA*), pDs‐ChgH‐vtg signal‐EGFP (Murakami et al. 2019) was digested with Asp718I and NotI. An insert fragment containing firefly *luciferase* (*LUC*) and *enhanced green fluorescent protein* (*EGFP*) was amplified from the plasmid pCS2‐LUC‐EGFP‐pA (Murakami et al. 2022) using PCR with a primer pair (Asp718I‐LUC‐FW and EGFP‐NotI‐RV) and digested with Asp718I and NotI. This fragment was ligated to the backbone to construct pDs‐ChgH‐LUC‐EGFP. (b) Construction of plasmids containing promoters that induce high expression. To generate another backbone fragment containing 5′‐and 3′‐*Ds* elements, *LUC*, *EGFP*, and *pA*; pDs‐ChgH‐LUC‐EGFP was digested with XhoI and Asp718I. Each insert fragment containing the *actb* or *ef1αA* promoter (Hamada et al. 1998; Kinoshita et al. 1999) was amplified from genomic DNA using PCR with a primer pair (SalI‐actb‐FW/actb‐Asp718I‐RV or XhoI‐ef1αA‐FW/ef1αA‐Asp718I‐RV) and digested with SalI (for *actb*) or XhoI (for *ef1αA*) and Asp718I. Each insert fragment was ligated to the backbone to construct pDs‐Actb‐LUC‐EGFP or pDs‐Ef1αA‐LUC‐EGFP. (c) Construction of plasmids containing *nt5* genes. To evaluate IMP degradation activity, three plasmids expressing the fusion protein of Nt5 and the reporter protein were generated. The backbone containing 5′‐and 3′‐*Ds* elements, *LUC*, *EGFP* and *pA* was amplified from the plasmid pDs‐Ef1αA‐LUC‐EGFP using a primer pair (Backbone‐FW/Backbone‐RV) via PCR. The insert containing *nt5ea* was amplified from the plasmid pCS2‐nt5ea‐LUC‐EGFP using primer pair (Insert‐nt5ea‐FW/Insert‐nt5ea‐RV) via PCR (Murakami et al. 2022). The inserts containing *nt5c1aa* or *nt5c1ab* were amplified from muscle or liver tissue‐derived cDNA using primers (Insert‐nt5c1aa‐FW/Insert‐nt5c1aa‐RV or Insert‐nt5c1ab‐FW/Insert‐nt5c1ab‐RV) via PCR. Each insert fragment possessed homologous sequences (HSs) at both ends that matched the backbone fragment's junctions. The plasmids pDs‐Ef1αA‐nt5ea‐LUC‐EGFP, pDs‐Ef1αA‐nt5c1aa‐LUC‐EGFP, and pDs‐Ef1αA‐nt5c1ab‐LUC‐EGFP were constructed by connecting each insert fragment to the backbone fragment using the In‐Fusion cloning method. (d) Construction of the *nt5c1aa* L305P mutant plasmid. To generate the *nt5c1aa* L305P mutant, a single amino acid substitution (Leu → Pro) was introduced into the nt5c1aa coding sequence. The mutation was designed by changing the codon CTG (Leu) to CCC (Pro) at the target position. Site‐directed mutagenesis was performed using two sets of primers (nt5c1aa‐L305P‐FW1/nt5c1aa‐L305P‐RV2 or nt5c1aa‐L305P‐FW2/nt5c1aa‐L305P‐RV1), which introduced HSs containing the desired nucleotide substitution. Substituted nucleotides (CC) are indicated by red stars. Resulting mutant fragments were connected using the In‐Fusion cloning method to construct pDs‐Ef1αA‐nt5c1aa‐L305P‐LUC‐EGFP.
**Figure S2:** Dose‐dependent effects of plasmid DNA injection on embryo survival and transgene expression. (a) Survival rates of embryos injected with plasmid DNA at concentrations of 1, 10, or 20 ng/μL into the yolk. Numbers within bars indicate numbers of surviving (gray) and dead (black) embryos; total numbers of injected embryos (n) are shown above each bar. Different letters indicate significant differences among groups according to Fisher's exact test. (b) Luciferase activity measured at 1 dpf following yolk injection of plasmid DNA at indicated concentrations. Values are shown as fold change relative to the 1 ng/μL group. Data are presented as mean ± SD. Different letters indicate significant differences among groups according to one‐way ANOVA followed by Tukey's HSD test.
**Figure S3:** Nucleotide sequence alignment of *nt5c1aa* (a) and *nt5c1ab* (b) from the Cab and Hd‐rR strains. The displayed regions correspond to the mutated portions extracted from the full‐length coding sequences. The asterisks below each alignment denote identical bases, while mismatched nucleotides are shown in white text on a black background. Silent substitutions are marked by pink lines, and missense mutations are highlighted with green outlines. Nucleotide positions are indicated by the numbers at both ends of each line.
**Figure S4:** In silico prediction of signal peptides in medaka Nt5c1aa and Nt5c1ab using SignalP. A putative cleavage site (CS) was detected between residues 21 and 22 in Nt5c1aa (a), whereas no signal peptide was predicted for Nt5c1ab (b). SignalP identifies potential secretory signal peptides that are translocated via the Sec pathway and cleaved by Signal Peptidase I (Sec/SPI). Probabilities corresponding to the N‐terminal (Sec/SPI n), central hydrophobic (Sec/SPI h), and C‐terminal (Sec/SPI c) regions of the signal peptide are shown as red, orange, and yellow lines, respectively, whereas probabilities for non‐signal peptide regions (OTHER) are represented by pink dashed lines.
**Figure S5:** Measurement of LUC activity in embryos injected with plasmids harboring nt5ea‐LUC‐EGFP, nt5c1aa‐LUC‐EGFP, or nt5c1ab‐LUC‐EGFP constructs. LUC activity levels were normalized to those of the nt5ea‐LUC‐EGFP group, which was arbitrarily defined as “1”. Data represent the mean ± SD from three independent experiments (*n* = 40 per group). No statistically significant differences were observed among the groups, as determined using one‐way ANOVA followed by Tukey's HSD test.
**Figure S6:** Validation of IMP normalization using total protein content. (a) Time‐course changes in IMP levels normalized to total protein content in embryos expressing nt5ea‐LUC‐EGFP, nt5c1aa‐LUC‐EGFP, or nt5c1ab‐LUC‐EGFP. Different letters indicate statistically significant differences among groups at each time point according to one‐way ANOVA followed by Tukey's HSD test. (b) Total protein levels in embryos expressing each nt5‐LUC‐EGFP construct. Values are shown as fold change relative to nt5ea‐LUC‐EGFP. Data are presented as mean ± SD.
**Figure S7:** Structural analysis of wild‐type and mutant Nt5c1aa using AlphaFold2. (a) Predicted structure of wild‐type Nt5c1aa protein. Colors indicate local confidence scores (pLDDT): blue (> 90), cyan (90–70), yellow (70–50), and orange (< 50). (b) Predicted structure of L305P mutant Nt5c1aa protein. The pLDDT color scheme is the same as in (a). (c) Superposition of wild‐type (gray) and mutant (magenta) Nt5c1aa structures, focusing on motif S. Motif S in the wild‐type protein is shown in orange, whereas the corresponding region in the mutant protein is shown in green. The substitution induces a local conformational difference without altering the overall fold. (d) Superposition of wild‐type and mutant Nt5c1aa structures focusing on the amino acid residue at position 305. The side chain of 305 L in the wild‐type protein (orange) and 305P in the mutant protein (green) adopt distinct orientations, suggesting potential effects on substrate recognition.
**Figure S8:** Functional comparison of IMP degradation between wild‐type Nt5c1aa and the L305P mutant. (a) Time‐course analysis of IMP levels in embryos expressing Nt5c1aa or Nt5c1aa‐L305P. IMP amounts were measured at the indicated time points without normalization to reporter activity. The L305P mutant exhibited significantly lower IMP levels than wild‐type Nt5c1aa at 3 h, whereas no significant differences were detected at earlier time points (0–2 h), indicating a modest enhancement of IMP‐degrading activity at later stages. The asterisk indicates that the values are significantly different between the groups according to one‐way ANOVA followed by Tukey's HSD test. *p < 0.05. (b) Luciferase activity of embryos expressing Nt5c1aa‐LUC‐EGFP or Nt5c1aa‐L305P‐LUC‐EGFP. Reporter activity did not differ significantly between groups, confirming comparable expression levels of the fusion proteins. Statistical significance was determined using Welch's *t*‐test. Data are presented as mean ± SD of three measurements from different enzyme preparations.

**Table S1:** Reaction rates of Nt5 family enzymes.
**Table S2:** Comparison of reaction rates between Nt5c1aa and Nt5c1aa‐L305P.
**Table S3:** Oligonucleotide sequences used in this study.

## Data Availability

Plasmids with the *Ds* sequences used in this study will be distributed from RIKEN Bioresource Center DNA Bank (https://dna.brc.riken.jp/en/).
