# Supplementary material for: Efficient Gene Expression System in Medaka Embryos Enables Functional Characterization of nt5c1a Paralogs Involved in Inosine Monophosphate Metabolism
Source: Genesis. 2026 May 9;64:e70056. doi: 10.1002/dvg.70056 (PMC13156797; doi:10.1002/dvg.70056)
Supplement: Supplementary file 1 — Figure S1: Schematic diagram of the plasmids used. To induce transposon‐mediated high expression in medaka embryos, plasmids with different promoters were constructed using a two‐step process (a, b). (a) Construction of the plasmid containing the reporter gene. To obtain a backbone fragment containing 5′‐and 3′‐Ds elements and an SV40 polyA signal (pA), pDs‐ChgH‐vtg signal‐EGFP (Murakami et al. 2019) was digested with Asp718I and NotI. An insert fragment containing firefly luciferase (LUC) and enhanced green fluorescent protein (EGFP) was amplified from the plasmid pCS2‐LUC‐EGFP‐pA (Murakami et al. 2022) using PCR with a primer pair (Asp718I‐LUC‐FW and EGFP‐NotI‐RV) and digested with Asp718I and NotI. This fragment was ligated to the backbone to construct pDs‐ChgH‐LUC‐EGFP. (b) Construction of plasmids containing promoters that induce high expression. To generate another backbone fragment containing 5′‐and 3′‐Ds elements, LUC, EGFP, and pA; pDs‐ChgH‐LUC‐EGFP was digested with XhoI and Asp718I. Each insert fragment containing the actb or ef1αA promoter (Hamada et al. 1998; Kinoshita et al. 1999) was amplified from genomic DNA using PCR with a primer pair (SalI‐actb‐FW/actb‐Asp718I‐RV or XhoI‐ef1αA‐FW/ef1αA‐Asp718I‐RV) and digested with SalI (for actb) or XhoI (for ef1αA) and Asp718I. Each insert fragment was ligated to the backbone to construct pDs‐Actb‐LUC‐EGFP or pDs‐Ef1αA‐LUC‐EGFP. (c) Construction of plasmids containing nt5 genes. To evaluate IMP degradation activity, three plasmids expressing the fusion protein of Nt5 and the reporter protein were generated. The backbone containing 5′‐and 3′‐Ds elements, LUC, EGFP and pA was amplified from the plasmid pDs‐Ef1αA‐LUC‐EGFP using a primer pair (Backbone‐FW/Backbone‐RV) via PCR. The insert containing nt5ea was amplified from the plasmid pCS2‐nt5ea‐LUC‐EGFP using primer pair (Insert‐nt5ea‐FW/Insert‐nt5ea‐RV) via PCR (Murakami et al. 2022). The inserts containing nt5c1aa or nt5c1ab were amplified from muscle or liver t [file DVG-64-e70056-s002.pdf]

Supplementary Fig. 1 Murakami *et al.*

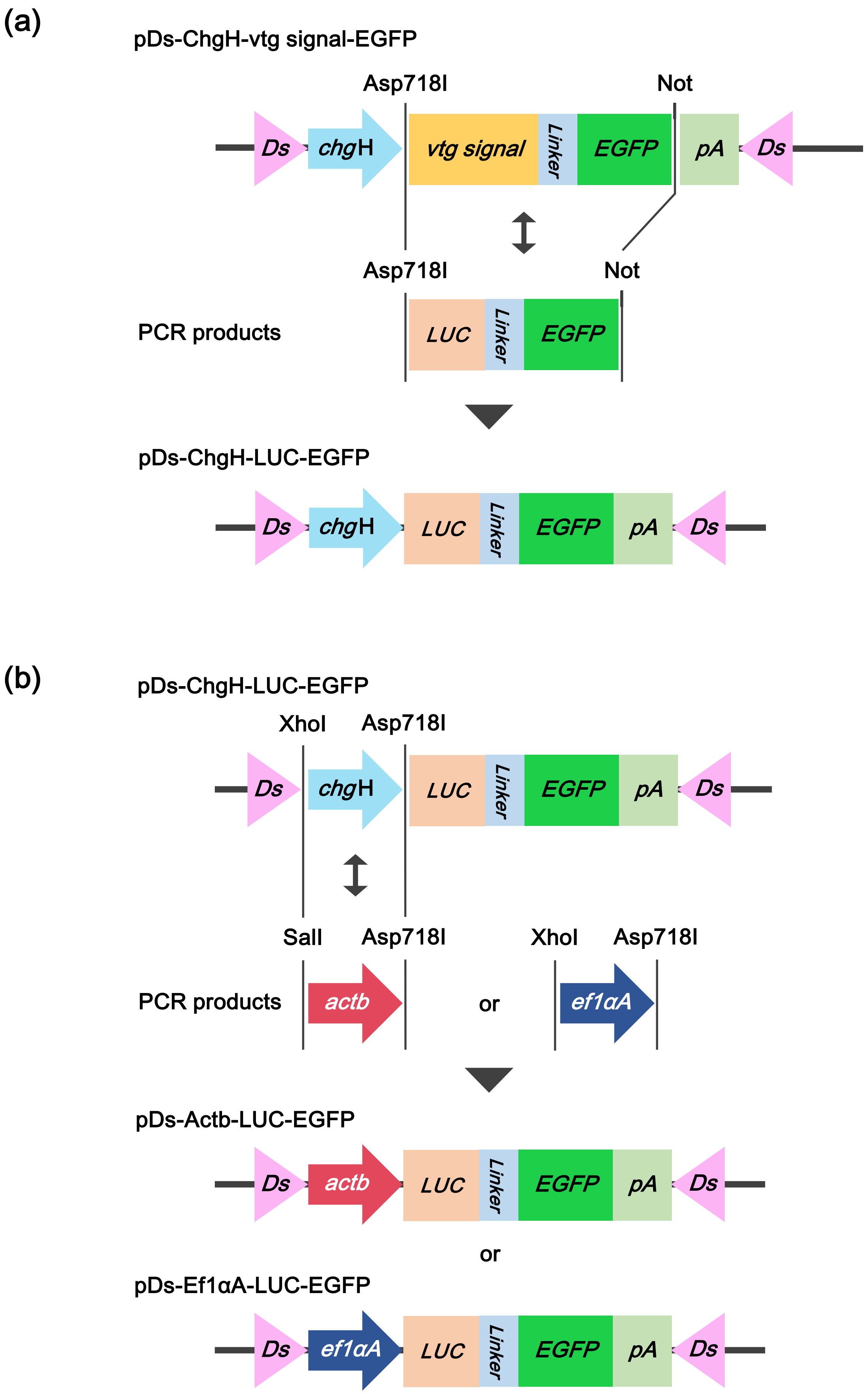

(c)

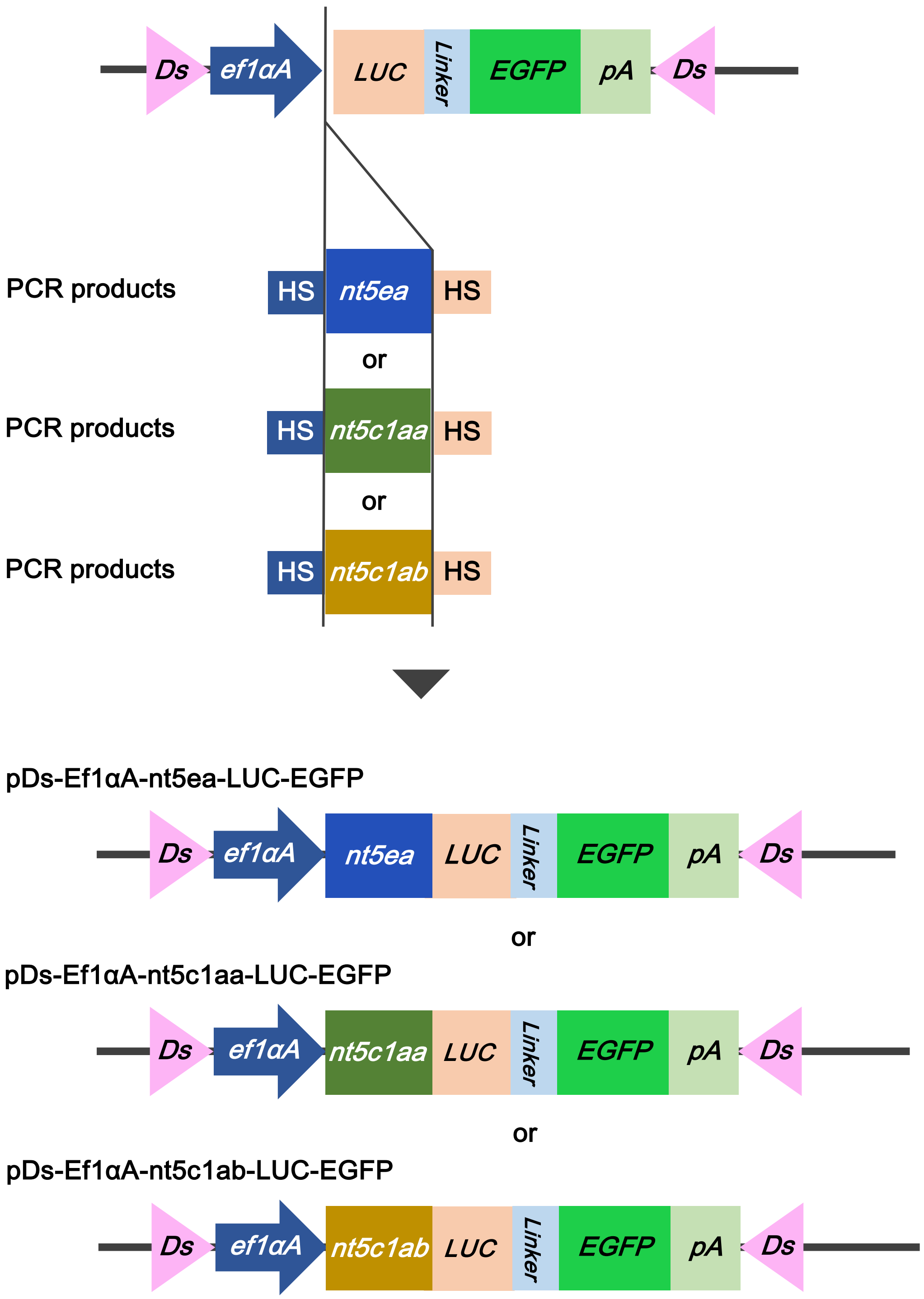
Ds
ef1αA
LUC
Linker
EGFP
pA
Ds
PCR products
HS
nt5ea
HS
or
PCR products
HS
nt5c1aa
HS
or
PCR products
HS
nt5c1ab
HS
pDs-Ef1αA-nt5ea-LUC-EGFP
Ds
ef1αA
nt5ea
LUC
Linker
EGFP
pA
Ds
or
pDs-Ef1αA-nt5c1aa-LUC-EGFP
Ds
ef1αA
nt5c1aa
LUC
Linker
EGFP
pA
Ds
or
pDs-Ef1αA-nt5c1ab-LUC-EGFP
Ds
ef1αA
nt5c1ab
LUC
Linker
EGFP
pA
Ds

(d)

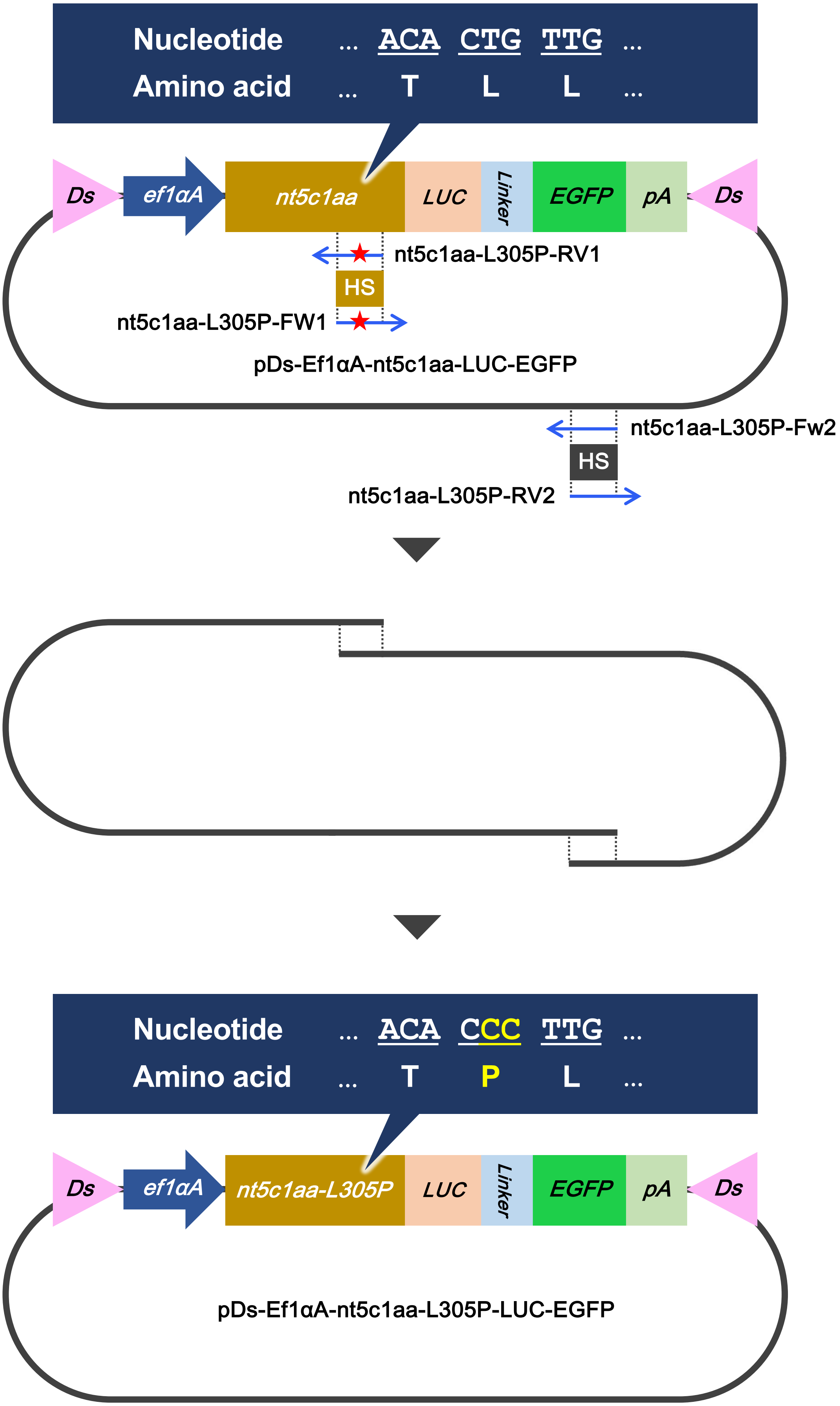
Nucleotide ... ACA CTG TTG ...
Amino acid ... T L L ...
Ds
ef1αA
nt5c1aa
LUC
Linker
EGFP
pA
Ds
nt5c1aa-L305P-RV1
HS
nt5c1aa-L305P-FW1
pDs-Ef1αA-nt5c1aa-LUC-EGFP
nt5c1aa-L305P-Fw2
HS
nt5c1aa-L305P-RV2
Nucleotide ... ACA CCC TTG ...
Amino acid ... T P L ...
Ds
ef1αA
nt5c1aa-L305P
LUC
Linker
EGFP
pA
Ds
pDs-Ef1αA-nt5c1aa-L305P-LUC-EGFP

Supplementary Fig. 2

Murakami *et al.*

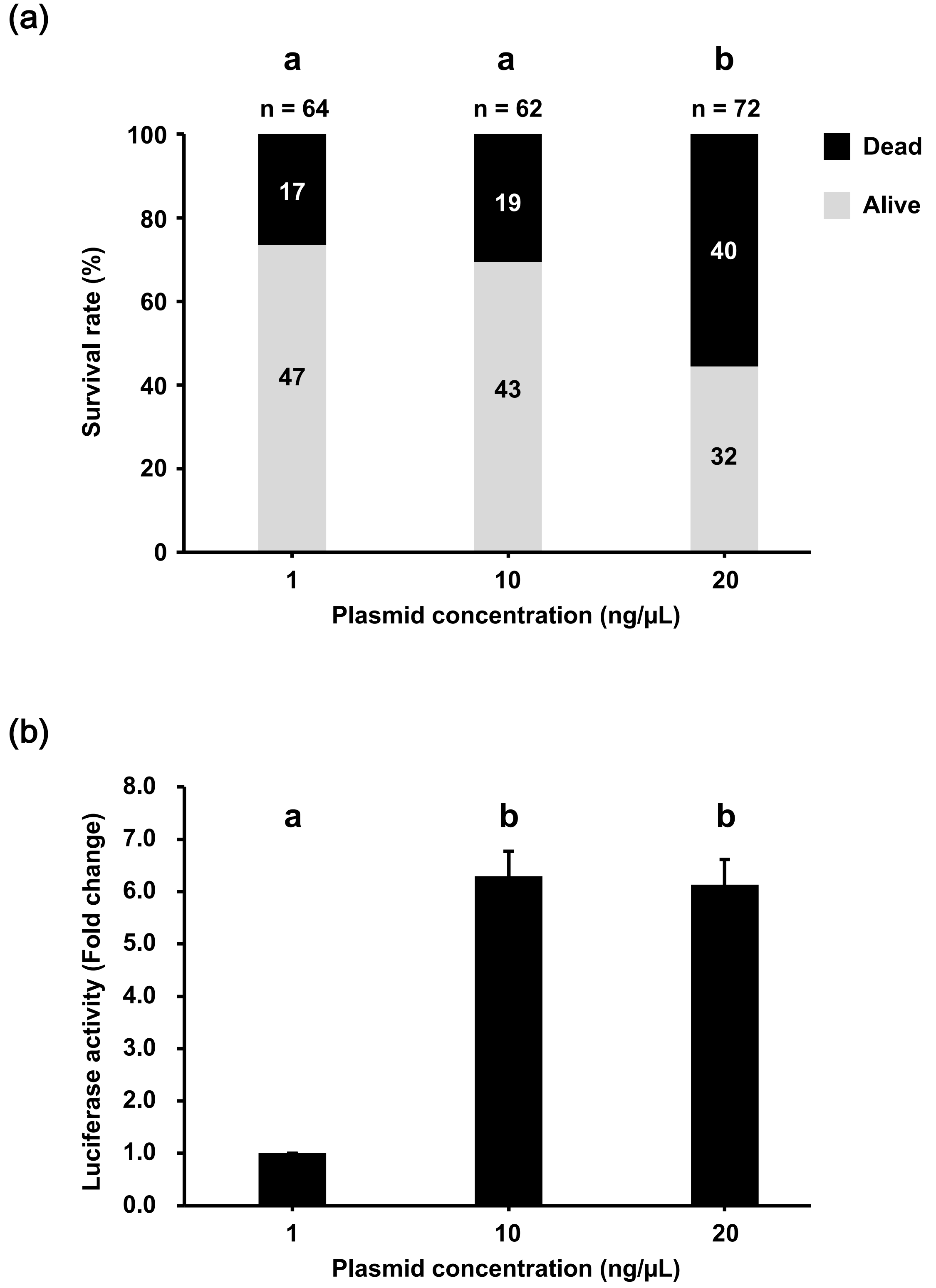

Supplementary Fig. 3

Murakami *et al.*

(a)

```
                                                         Gly
Cab         1 ATGCTGCAGACGGCAGCAGCTCTGTCTGTTTATTTGGTTGGAGGTTTTTTTTTTGTCTTA 60
Hd-rR       1 ATGCTGCAGACGGCAGCAGCTCTGTCTGTTTATTTGGTTGGAGGGTTTTTTTTTGTCTTA 60
              ******************************************** ***************

                                               Ala                  Ser
Cab       301 GAGGGAGCCGTGTTCTTTCCGAAGCCGGAGAACGCTGTGACGATCGCGGTTTCCTCCCGG 360
Hd-rR     301 GAGGGAGCCGTGTTCTTTCCGAAGCCGGAGAACGCCGTGACGATCGCGGTTTCCTCTCGG 360
              *********************************** ******************** ***

                                             Ala
Cab       961 CTCCAGAAGAAGTTTTATGCAAAAGGCCAGCGCCTGAACTGCCCCATTCGCACCTACCTG 1020
Hd-rR     961 CTCCAGAAGAAGTTTTATGCAAAAGGCCAGCGTCTGAACTGCCCCATTCGCACCTACCTG 1020
              ******************************** ***************************
                                             Val

                            Thr
Cab      1021 GTTACAGCTCGCAGTACAGCAAGCTCTGGGATTCGTGCGCTGAAGACGCTCAGGGCCTGG 1080
Hd-rR    1021 GTTACAGCTCGCAGTGCAGCAAGCTCTGGGATTCGTGCGCTGAAGACGCTCAGGGCCTGG 1080
              *************** ********************************************
                            Ala

                                      Phe            Pro
Cab      1081 GGACTGGAGGTGGACGAGGCCTTGTTCCTTGCAGGTGCACCTAAAGGTCCCATGCTAGAG 1140
Hd-rR    1081 GGACTGGAGGTGGACGAGGCCTTGTTTCTTGCAGGTGCACCAAAAGGTCCCATGCTAGAG 1140
              ************************** ************** ******************

                                      Asp
Cab      1141 AAAATCCGACCGCACATCTTCTTTGACGACCAGATGTTTCATGTGGAGGGAGCTGCAGAG 1200
Hd-rR    1141 AAAATCCGACCGCACATCTTCTTTGATGACCAGATGTTTCATGTGGAGGGAGCTGCAGAG 1200
              ************************** *********************************
```

(b)

```
                                      Glu
Cab       181 AGAGTTCTCTTCAACATGGAGAAGGAACAGCAGATCTTTGAACAGCAGGGCATGGAGGAG 240
Hd-rR     181 AGAGTTCTCTTCAACATGGAGAAGGAGCAGCAGATCTTTGAACAGCAGGGCATGGAGGAG 240
              ************************** *********************************

                                         Thr
Cab       241 TACATCAAGTACCAGGTGGCGCATGAAACTGAGTCTTTCAGTCCTGGACCGGCCTTCTCT 300
Hd-rR     241 TACATCAAGTACCAGGTGGCGCATGAAACGGAGTCTTTCAGTCCTGGACCGGCCTTCTCT 300
              ***************************** ******************************

              Pro
Cab       481 CCCATCGGCTACCTGAAGGCCTACCACACTAATCTGTATCTGTCTGCTGATCCAGATAAA 540
Hd-rR     481 TCCATCGGCTACCTGAAGGCCTACCACACTAATCTGTATCTGTCTGCTGATCCAGATAAA 540
               ***********************************************************
              Ser

                    Glu
Cab       541 GTTCATGAGGCTCTAGAGGCAGGTATTGCAGCAGCCACCATGTTCACTCCAGACAAGATG 600
Hd-rR     541 GTTCATGAAGCTCTAGAGGCAGGTATTGCAGCAGCCACCATGTTCACTCCAGACAAGATG 600
              ******** ***************************************************

                                      Phe                               Ala
Cab       961 GAGAAGATCAGGCCACACATCTTCTTTGACGACCAGATGTTTCACGTGGAGGGAGCGGCG 1020
Hd-rR     961 GAGAAGATCAGGCCACACATCTTCTTCGACGACCAGATGTTTCACGTGGAGGGAGCGGCA 1020
              ************************** ********************************

                             Ala
Cab      1021 GAAATGGGGACAGTGGCATGTCACGTGCCCTACGGCGTTGCACAGAGAATTGTGAGGAAA 1080
Hd-rR    1021 GAAATGGGGACAGTGGCGTGTCACGTGCCCTACGGCGTTGCACAGAGAATTGTGAGGAAA 1080
              ***************** ******************************************
```

Supplementary Fig. 4 Murakami *et al.*

(a)

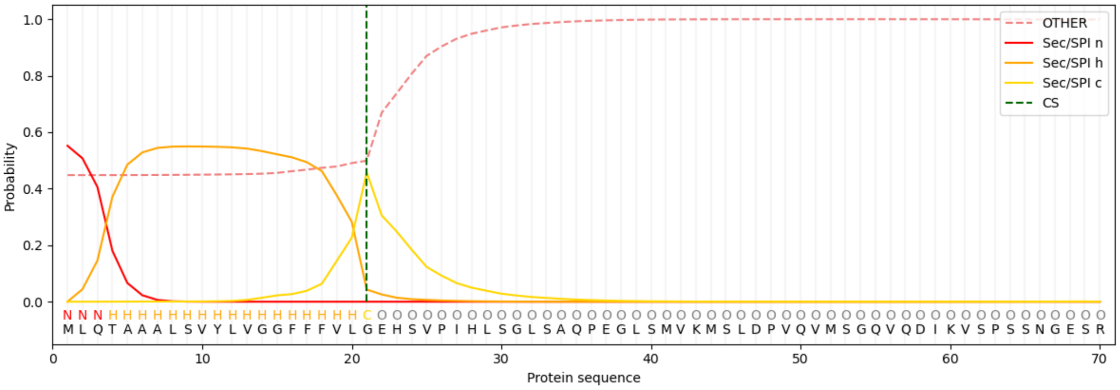

(b)

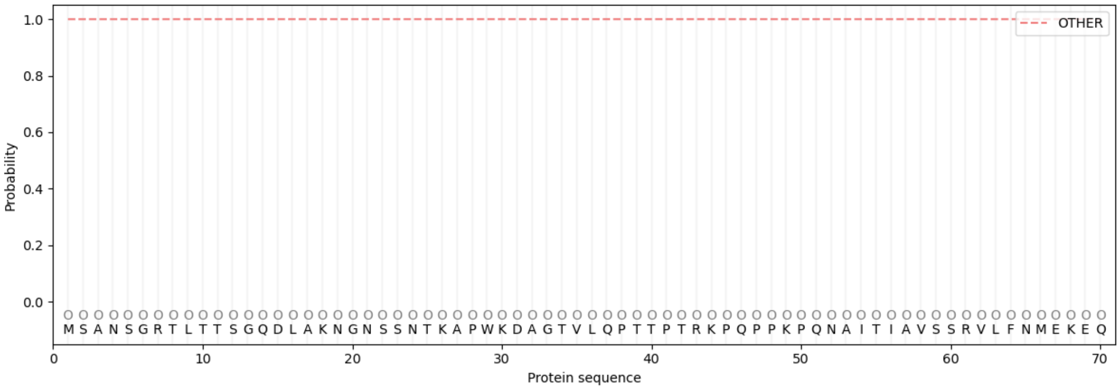

Supplementary Fig. 5

Murakami *et al.*

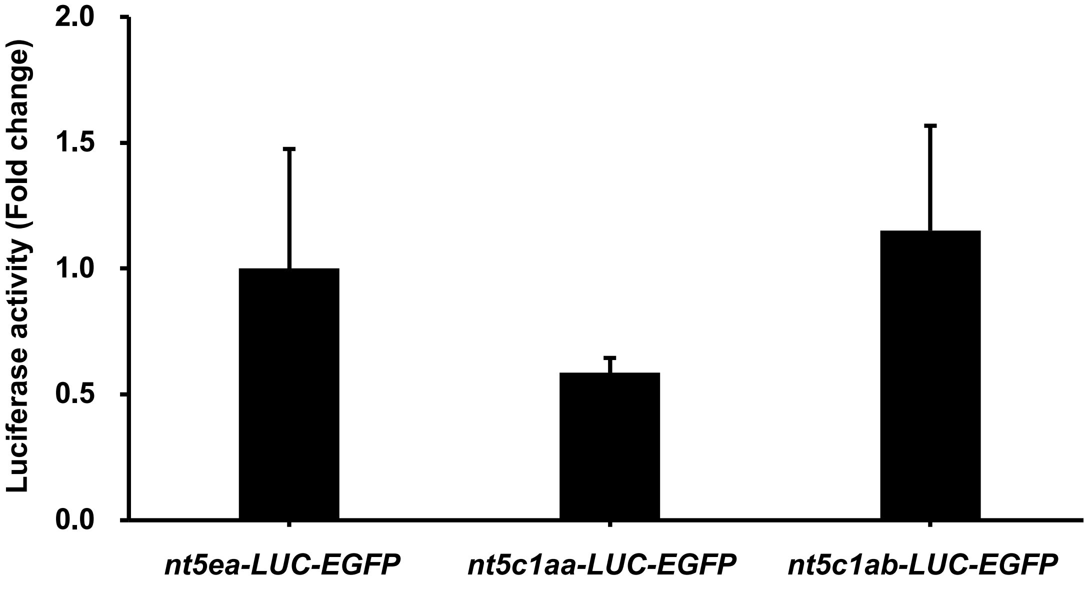

Supplementary Fig. 6

Murakami *et al.*

(a)

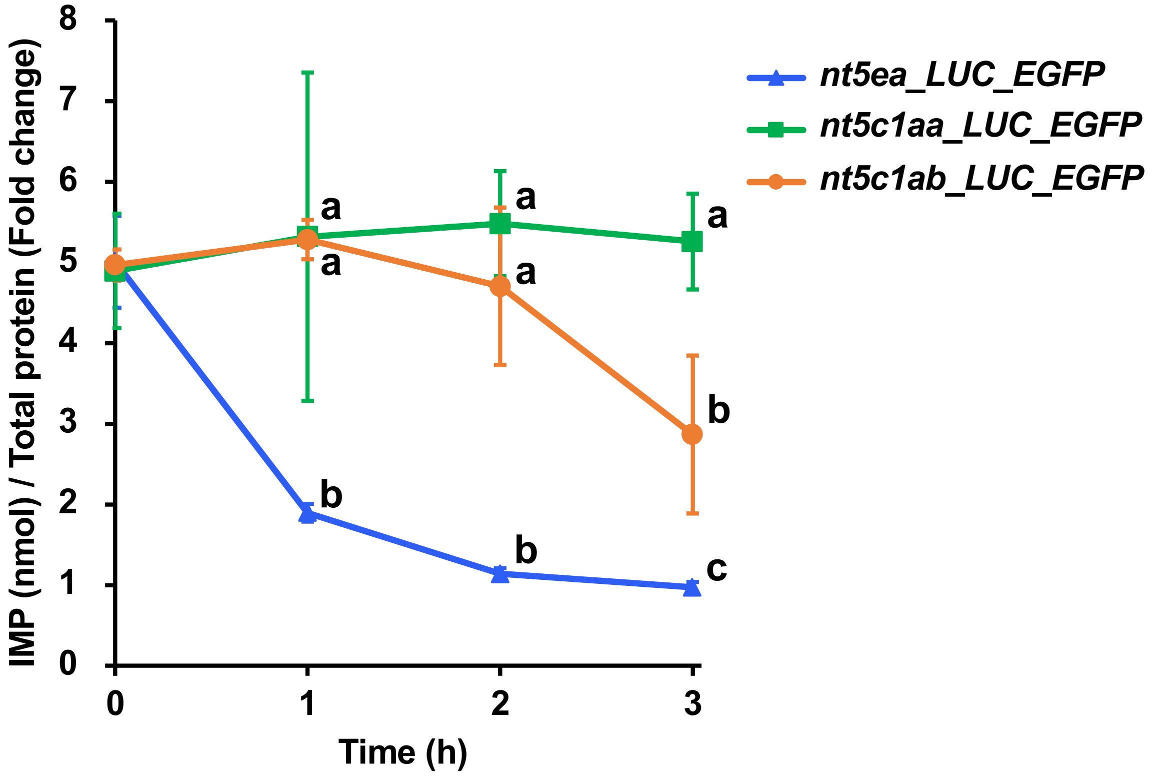

(b)

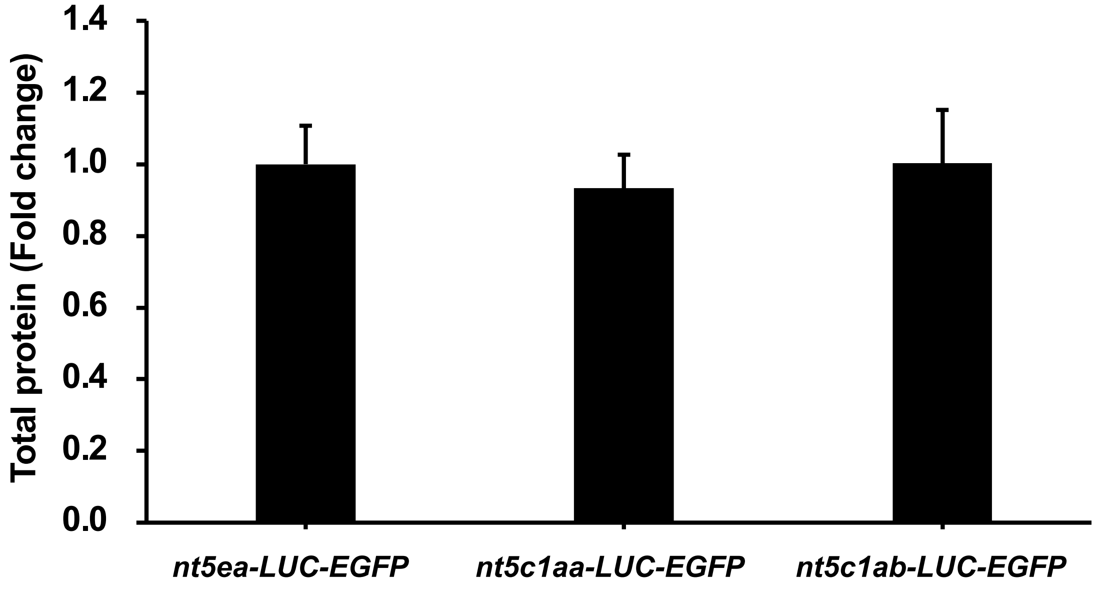

Supplementary Fig. 7

Murakami *et al.*

(a)

(b)

(c)

(d)

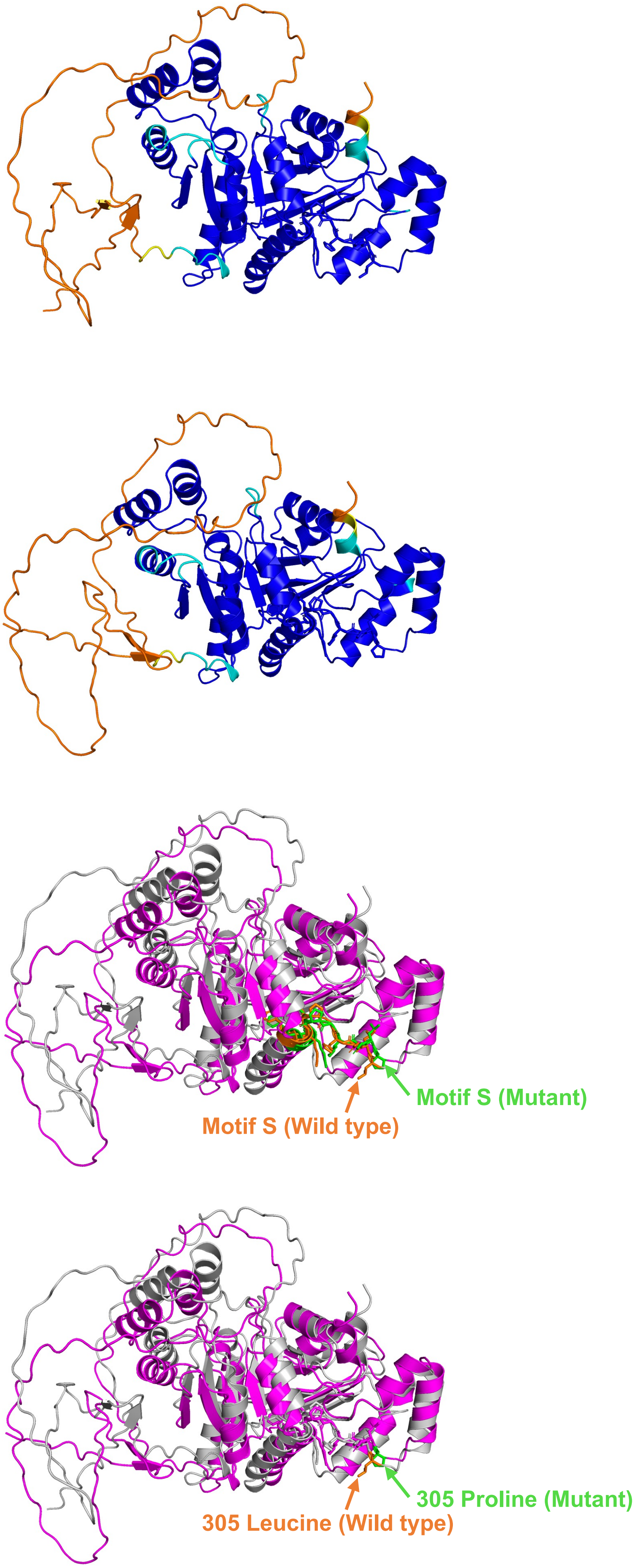

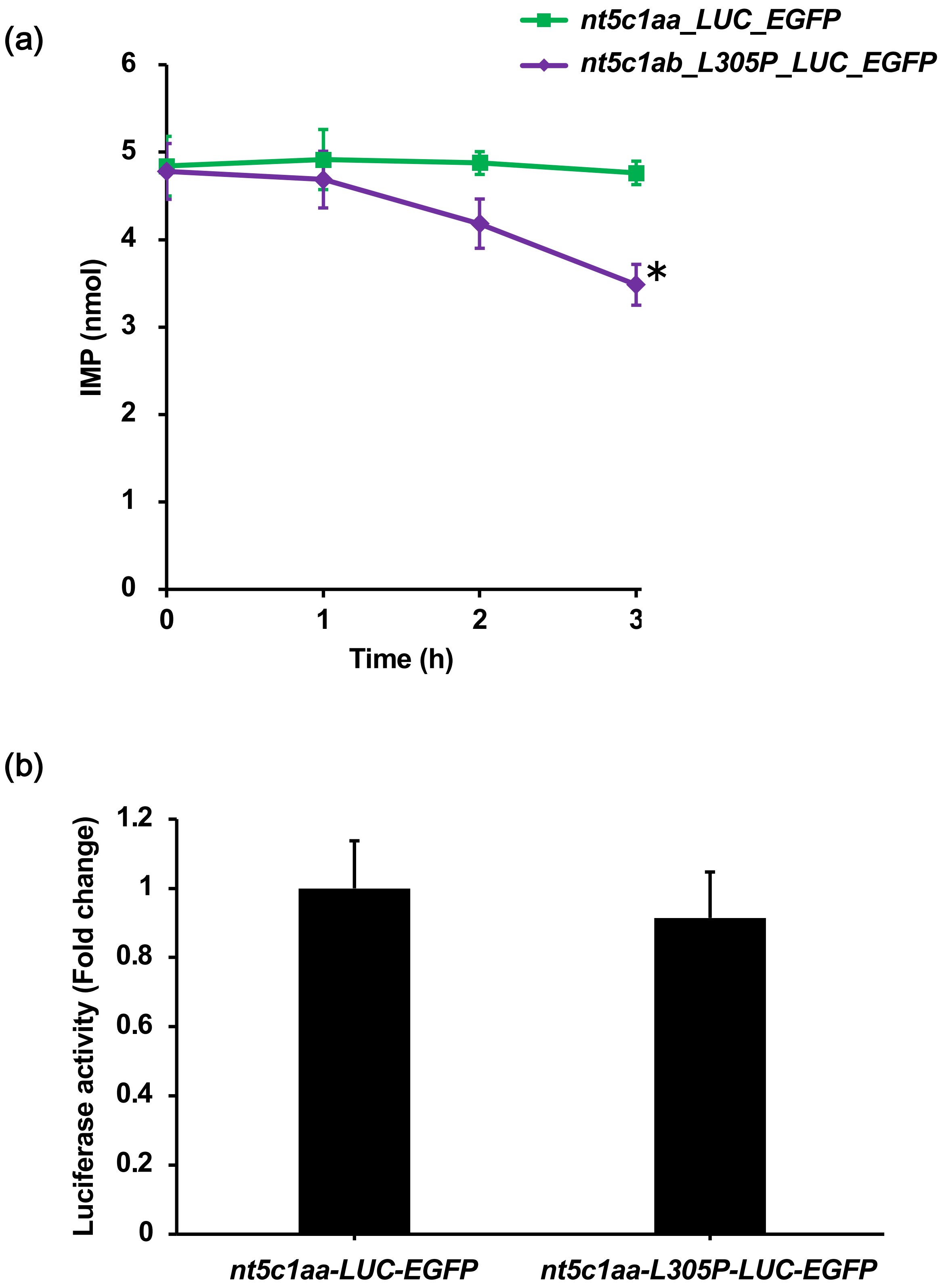
(a)
nt5c1aa_LUC_EGFP
nt5c1ab_L305P_LUC_EGFP
IMP (nmol)
6
5
4
3
2
1
0
*
0
1
2
3
Time (h)
(b)
Luciferase activity (Fold change)
1.2
1
0.8
0.6
0.4
0.2
0
nt5c1aa-LUC-EGFP
nt5c1aa-L305P-LUC-EGFP
