# Supplementary material for: Efficient Gene Expression System in Medaka Embryos Enables Functional Characterization of nt5c1a Paralogs Involved in Inosine Monophosphate Metabolism
Source: Genesis. 2026 May 9;64:e70056. doi: 10.1002/dvg.70056 (PMC13156797; doi:10.1002/dvg.70056)
Supplement: Supplementary file 2 — Table S1: Reaction rates of Nt5 family enzymes. Table S2: Comparison of reaction rates between Nt5c1aa and Nt5c1aa‐L305P. Table S3: Oligonucleotide sequences used in this study. [file DVG-64-e70056-s001.docx]

| **Supplementary Table 1**. Reaction rates of Nt5 family enzymes | | | |
| --- | --- | --- | --- |
| Group | Normalized IMP degradation rate (h⁻¹) | | |
| *nt5ea_LUC_EGFP* | -1.93 | ± | 0.28^a^ |
| *nt5c1aa_LUC_EGFP* | -0.04 | ± | 0.08^b^ |
| *nt5c1ab_LUC_EGFP* | -0.48 | ± | 0.20^b^ |
|  |  |  |  |
| Means with different letters are significantly different (*p*<0.05 , Tukey’s HSD test). | | | |

| **Supplementary Table 2**. Comparison of reaction rates between Nt5c1aa and Nt5c1aa-L305P | | | |
| --- | --- | --- | --- |
| Group | Normalized IMP degradation rate (h⁻¹) | | |
| *nt5c1aa_LUC_EGFP* | 0.02 | ± | 0.14 |
| *nt5c1aa_L305P_LUC_EGFP* | -0.32 | ± | 0.33 |
|  |  |  |  |
| No significant differences were observed between groups (*p*>0.05 , Welch’s t-test). | | | |

| **Supplementary Table 3**. Oligonucleotide sequences used in this study. | |
| --- | --- |
| Name | Sequence (5'-3') |
| Asp718I-LUC-FW | GTCGGTACCATGCATCATCATCATCATCATGGAGGTAGCGGTGGAACTACTAGTATGGAAGATGCCA |
| EGFP-NotI-RV | GACGCGGCCGCTTACTTGTACAGCTCGTCCATGCC |
| SalI-actb-FW | CTGGTCGACAATTACAGTGGTCATGAATATTATAT |
| actb-Asp718I-RV | TGCGGTACCGGCTAAACTGGAAAACAACAAACAAA |
| XhoI-ef1αA-FW | CTGCTCGAGGAAAACAAAGGGCCAGTCTAAATGTA |
| ef1αA-Asp718I-RV | GACGGTACCTTTGAATGTTTCTGTTCCAAAAGATA |
| Backbone-FW | GGTACCTTTGAATGTTTCTGTTCCAAAAG |
| Backbone-RV | GGAGGTAGCGGTGGAACTACTAGTATGG |
| Insert-nt5ea-FW | ACATTCAAAGGTACCATGACTCTCCGCTGGCGCTG |
| Insert-nt5ea-RV | TCCACCGCTACCTCCATGATGATGATGATGATGCATGCAGAGGTTCAGGAAAAG |
| Insert-nt5c1aa-FW | ACATTCAAAGGTACCATGCTGCAGACGGCAGCAGC |
| Insert-nt5c1aa-RV | TCCACCGCTACCTCCATGATGATGATGATGATGTTTCTTTTTGCTATGTAATTG |
| Insert-nt5c1ab-FW | ACATTCAAAGGTACCATGAGTGCAAACAGTGGCCG |
| Insert-nt5c1ab-RV | TCCACCGCTACCTCCATGATGATGATGATGATGCTTAACTACAGGAGAAGTCTC |
| nt5c1aa-L305P-FW1 | GACACACCCTTGGATCATGGTCCTTTTAAG |
| nt5c1aa-L305P-RV1 | GATCCAAGGGTGTGTCCTCGTTTTCCCTC |
| nt5c1aa-L305P-FW2 | ATTGCTGATAAATCTGGAGCCGGTG |
| nt5c1aa-L305P-RV2 | CCAGATTTATCAGCAATAAACCAGCCA |
